# Correction: Recruitment of RED-SMU1 Complex by Influenza A Virus RNA Polymerase to Control Ciral mRNA Splicing

**DOI:** 10.1371/journal.ppat.1004317

**Published:** 2014-07-23

**Authors:** The PLOS Pathogens Staff

## Notice of Republication

This article was republished on July 2, 2014, to correct a typographical error in the title that was introduced during the publication process. Please download this article again to view the correct version. The originally published, uncorrected article and the republished, corrected article are provided here for reference.

## Supporting Information

File S1 Originally published, uncorrected article
(PDF)

File S2 Republished, corrected article
(PDF)
