# Supplementary figures and images for: Recruitment of RED-SMU1 Complex by Influenza A Virus RNA Polymerase to Control Viral mRNA Splicing
Source: PLoS Pathog. 2014 Jun 12;10(6):e1004164. doi: 10.1371/journal.ppat.1004164 (PMC4055741; doi:10.1371/journal.ppat.1004164)

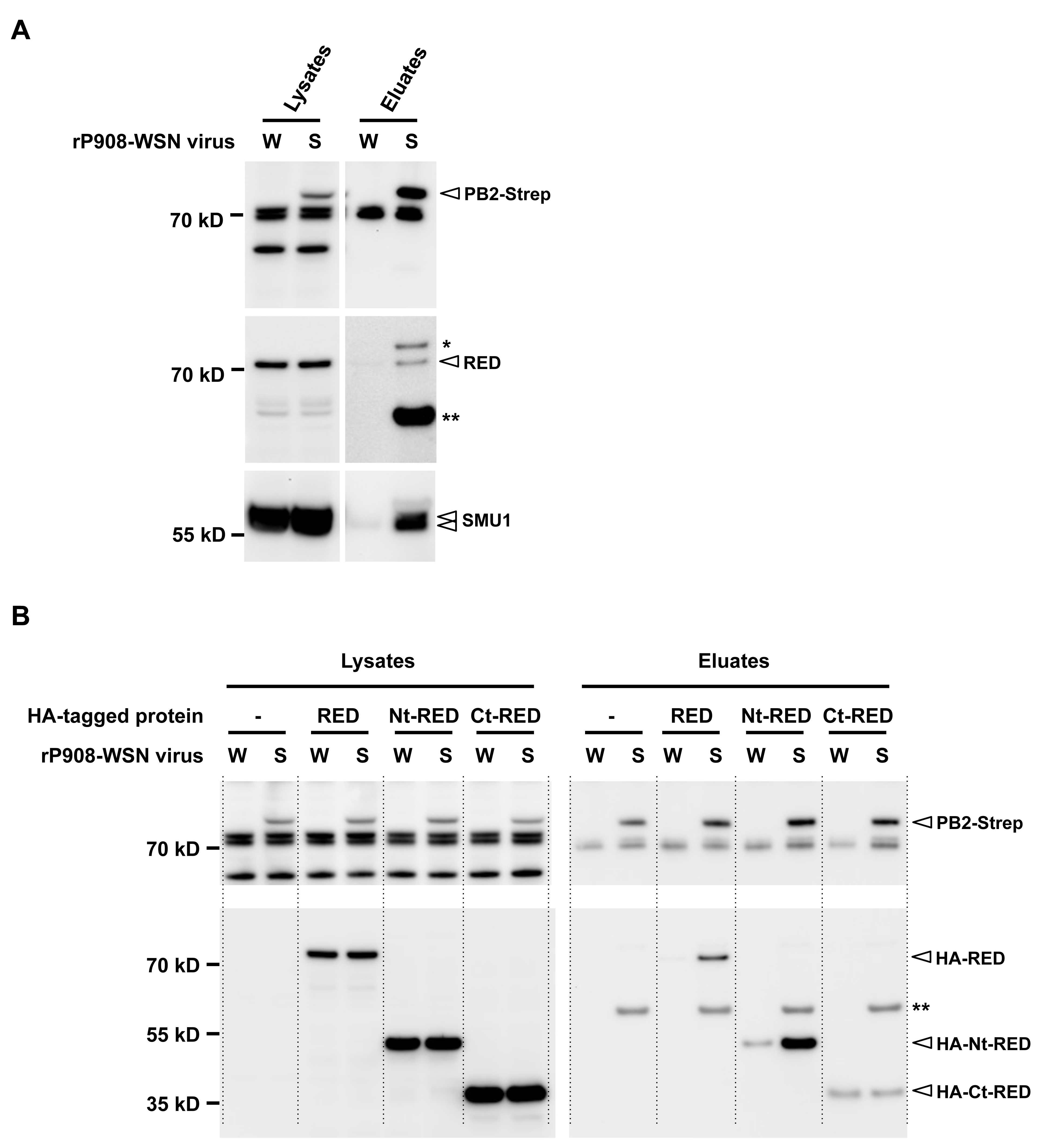

Supplement: Figure S1 — Co-purification of RED and SMU1 with P908 virus polymerase in infected cells. A. Co-purification of the endogenous RED and SMU1 proteins with the viral polymerase in infected cells. HEK-293T cells were infected at a m.o.i. of 5 with recombinant P908-WSN (W) or P908-Cstrep-WSN (S) viruses and incubated at 37°C for 6 hours. Whole-cell lysates were prepared and a fraction was incubated with StrepTactin beads as described in Material and Methods. Protein complexes were eluted, loaded on a 4–12% SDS-polyacrylamide gel and analyzed by western blotting using either StrepTactin to detect the PB2-Strep protein (upper panel) or an antibody specific for the RED or SMU1 protein (middle and lower panels). * and ** : non-specific detection of the PB2 and NP protein, respectively, as inferred from previous experiments [33]. B. Co-purification of the viral polymerase and recombinant, HA-tagged polypeptides corresponding to full-length or truncated forms of the RED protein. HEK-293T cells were transfected with the HA-RED, HA-Nt-RED or HA-Ct-RED expression plasmids, or mock-transfected with the pCI plasmid (−). 24 hours post-transfection, they were infected at a m.o.i. of 5 with recombinant P908-WSN (W) or P908-Cstrep-WSN (S) viruses and incubated at 37°C for 6 hours. Whole-cell lysates were prepared and a fraction was incubated with StrepTactin beads as described in Material and Methods. Protein complexes were eluted, loaded on a 4–12% SDS-polyacrylamide gel and analyzed by western blotting using either StrepTactin to detect the PB2-Strep protein (upper panel) or a monoclonal antibody specific for the HA tag (lower panel). **: non-specific detection of the NP protein, as inferred from western blot analysis using an anti-NP antibody. The bands detected in the PB2-Strep panel at 70 kD and the faster migrating band (present only in lysates) were also detected in mock-infected cells (data not shown). (TIF) [file ppat.1004164.s001.tif]

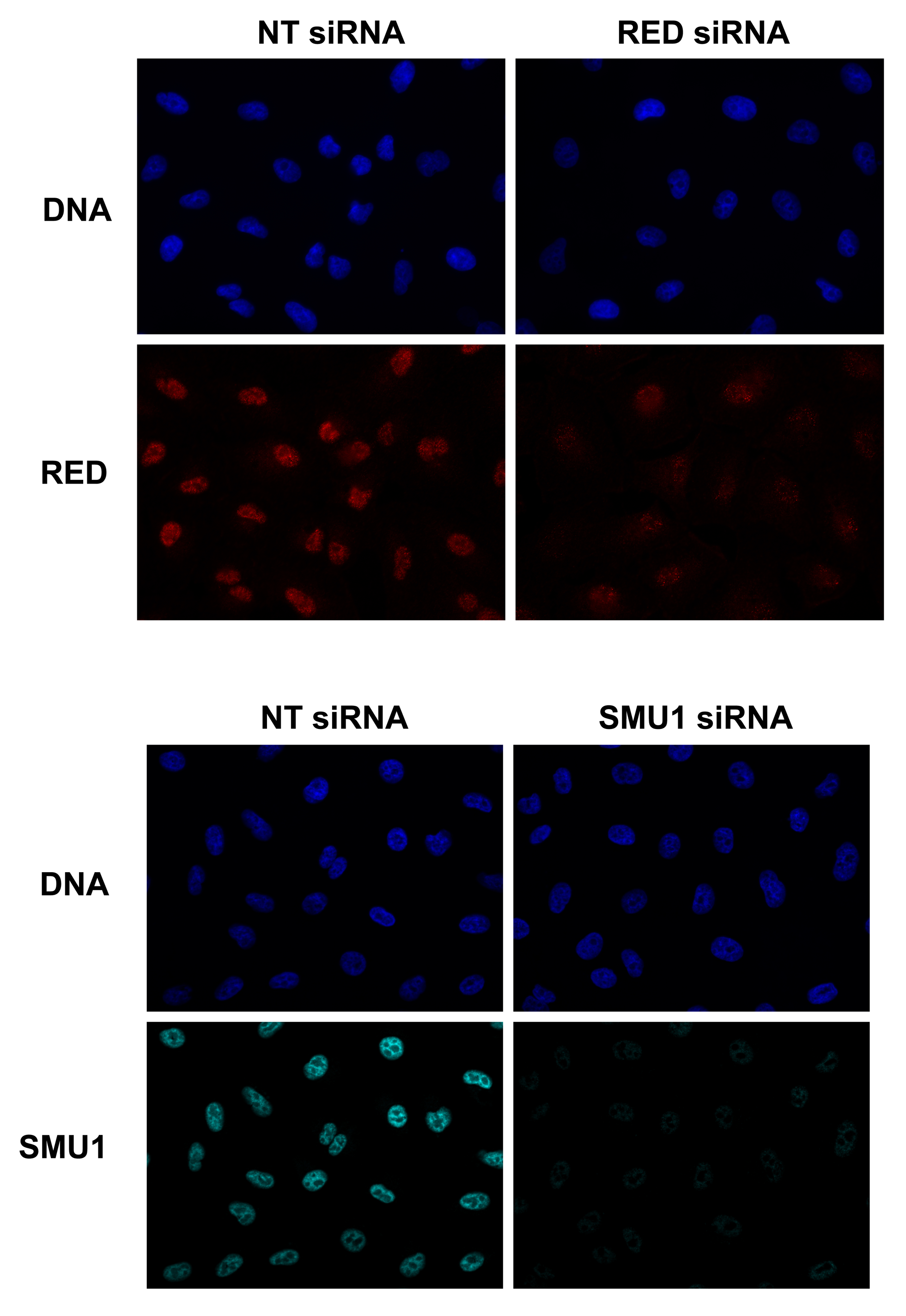

Supplement: Figure S2 — Immunofluorescence detection of RED and SMU1 in A549 cells treated with RED or SMU1 siRNAs. A549 cells were transfected with control non-target (NT), RED, or SMU1 siRNAs. At 41 hours post-transfection, cells were fixed, permeabilized, and stained with Hoechst 33342 and with an antibody specific for the RED (upper panels) or SMU1 protein (lower panels). Samples were analyzed under a fluorescence microscope (Inverted Zeiss Observer Z1). (TIF) [file ppat.1004164.s002.tif]

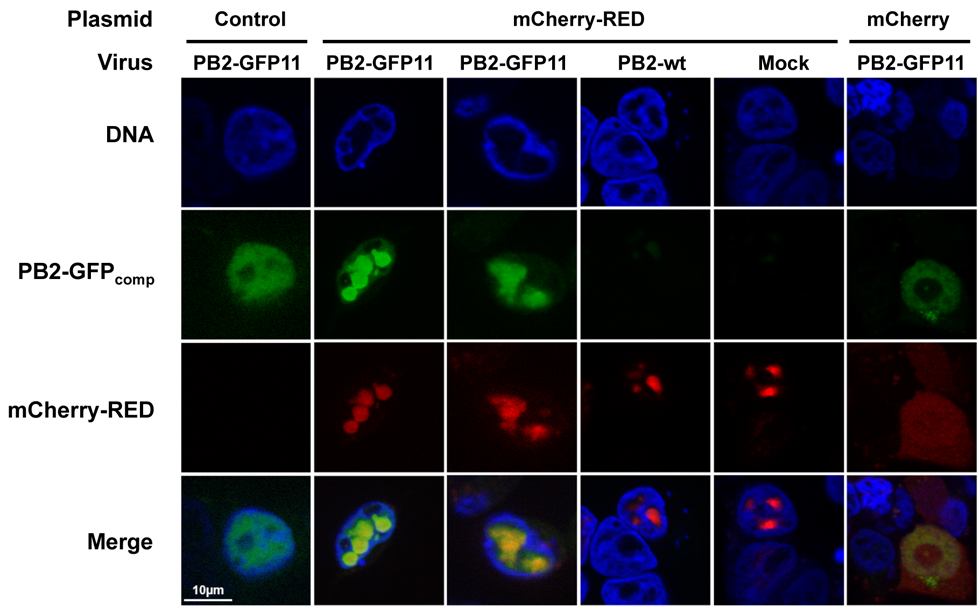

Supplement: Figure S3 — Relocalisation of PB2 with over-expressed mCherry-RED in infected cells. HEK-293T cells on coverslips were transfected with the pCMV-GFP1-10 plasmid together with the pCI-mCherry-RED, pCI-mCherry or control pCI plasmid, as indicated. At 24 hours post-transfection, they were infected with the rWSN (PB2-wt) or rWSN-PB2-GFP11 (PB2-GFP11) recombinant virus at a m.o.i. of 5 pfu/cell, or mock-infected. Cells were fixed at 6 hpi and they were stained with Hoechst 33342. Samples were analyzed under a fluorescence microscope (Inverted Zeiss Observer Z1). A merge of the signals corresponding to Hoechst-stained DNA (blue), PB2-GFPcomp (green) and mCherry-RED (red) is shown. A scale bar is shown. (TIF) [file ppat.1004164.s003.tif]

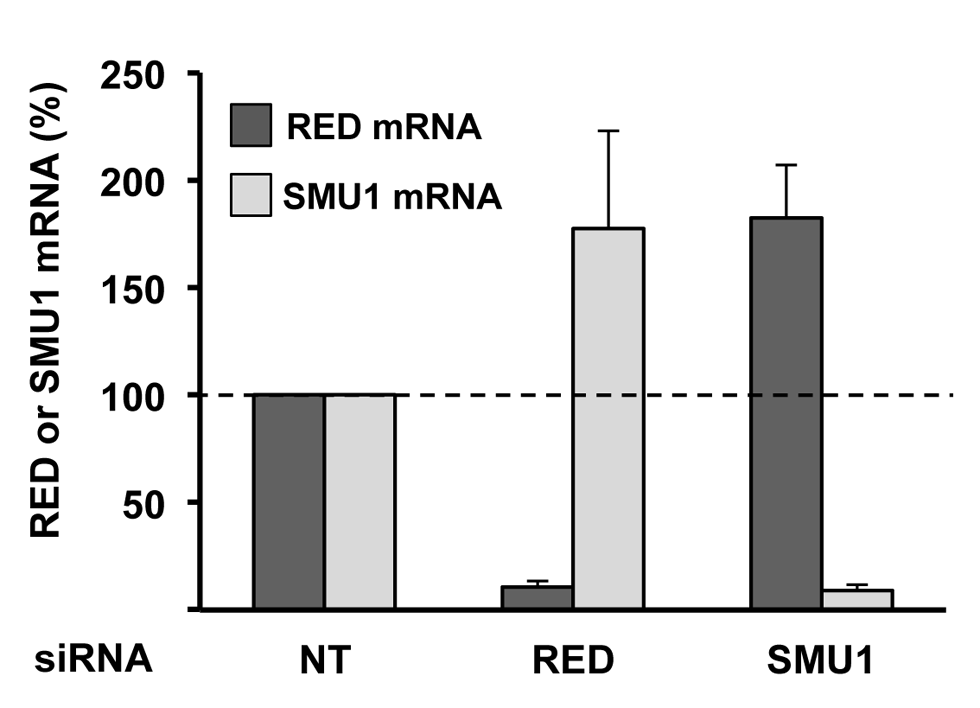

Supplement: Figure S4 — Quantification of RED and SMU1 mRNAs upon treatment of A549 cells with RED or SMU1 siRNAs. A549 cells were transfected with control siRNAs (NT), or with pools of four RED or SMU1 siRNAs. Total RNA was prepared at 36 hours post-transfection, polyA+ RNAs were isolated, and RT-qPCR was performed using primers and probes specific for RED or SMU1. The levels of RED and SMU1 mRNAs in RED/SMU1 siRNA-treated cells compared to control cells were determined using the ΔΔCt method. The data are expressed as the mean +/− SD of quadruplicates. (TIF) [file ppat.1004164.s004.tif]

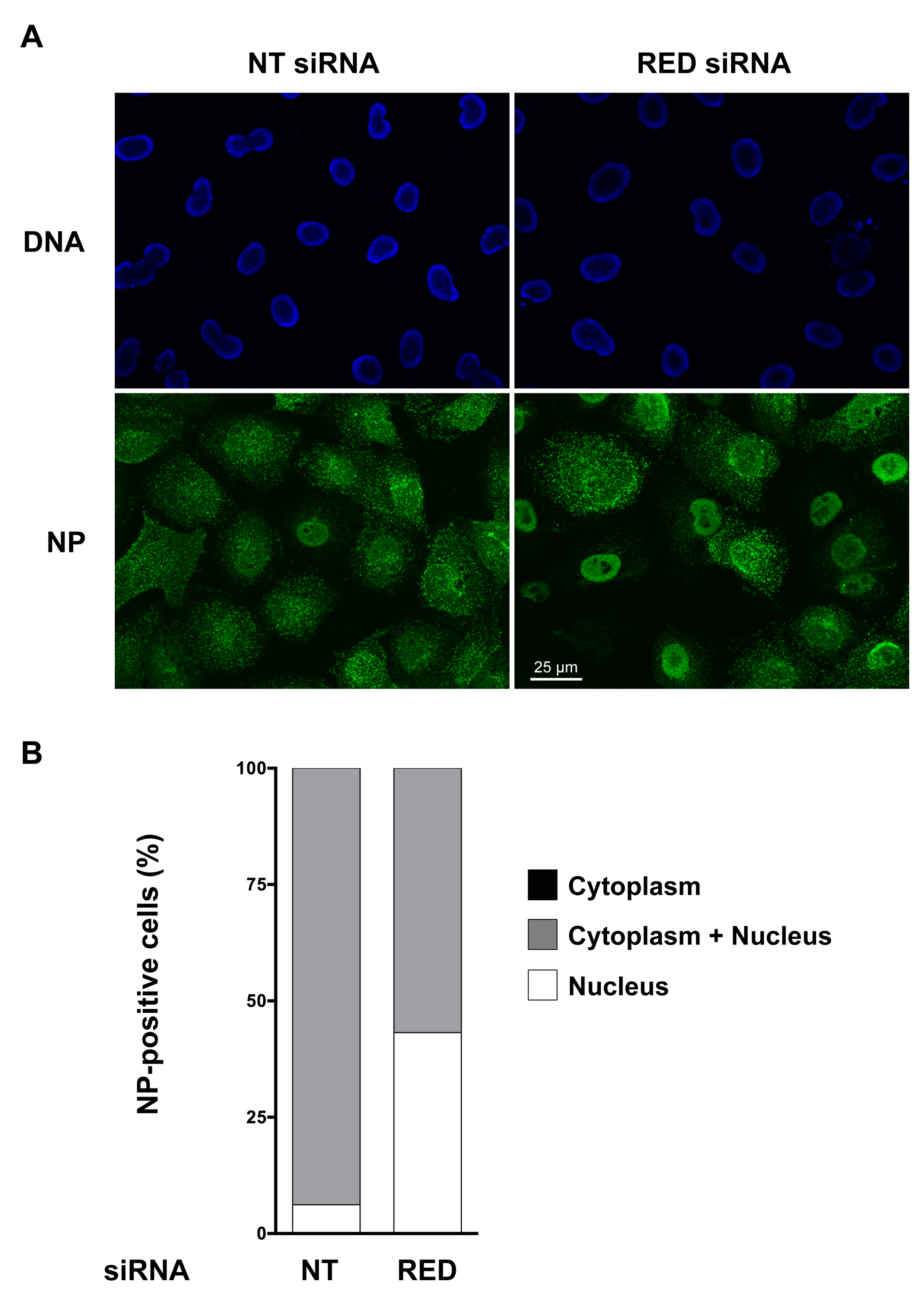

Supplement: Figure S5 — Effect of RED knock-down on the subcellular localisation of NP in P908-WSN influenza virus infected cells. A549 cells were transfected with control non-target (NT) or RED siRNAs, and were subsequently infected with the P908-WSN influenza virus at a m.o.i. of 5 pfu/cell. At 5 hpi, cells were fixed, permeabilized, and stained with an antibody specific for the NP protein and with Hoechst 33324. Samples were analyzed under a fluorescence microscope (Inverted Zeiss Observer Z1). A. Representative images of NP localization. B. Percentage of cells with different NP localization, based on the scoring of 145 and 132 cells for the NT and RED siRNA experimental condition, respectively. (TIF) [file ppat.1004164.s005.tif]
