# Supplementary material for: Recruitment of RED-SMU1 Complex by Influenza A Virus RNA Polymerase to Control Viral mRNA Splicing
Source: PLoS Pathog. 2014 Jun 12;10(6):e1004164. doi: 10.1371/journal.ppat.1004164 (PMC4055741; doi:10.1371/journal.ppat.1004164)
Supplement: Methods S1 — Supporting information is provided on the yeast two-hybrid-plus-one assay, indirect immunofluorescence assays and reverse transcription-quantitative PCR assays. (DOCX) [file ppat.1004164.s006.docx]

**Methods S1**

**Y2H +1 assay**

The viral ORFs were cloned into the pADH-NLS-Ade2 vector (Yves Jacob, unpublished) to express native proteins fused to a yeast nuclear localization signal, and into pDEST32-gw or pGBKT7-gw (modified, Gateway-compatible versions of pDEST32 and pGBKT7 plasmids from Invitrogen) to express Gal4-DB fusion proteins. The pDEST32-gw and pGBKT7-gw were used to screen the spleen and fetal brain cDNA library, respectively. Combinations of p3xH + pDEST32 or p3xH + pGBKT7 plasmids were transformed in AH109 strain (bait strain, Clontech). Y2H+1 assay was performed by yeast mating. Briefly, the human spleen and fetal brain cDNA libraries (Invitrogen) were cloned into the pPC86 plasmid (Invitrogen) and were transformed in Y187 strain (prey strain, Clontech). Each bait strain was mated with the library prey strain. Diploids were plated on synthetic defined medium lacking tryptophan, leucine, adenine, and histidine and supplemented with 3-aminotriazole (SD-W-L-A-H+3-AT). Positive clones were replica-platted during three weeks on selective medium to eliminate potential false-positives. AD-cDNAs (where AD refers to the Gal4 activation domain fused to human cDNAs) were PCR amplified from zymolase-treated yeast colonies and sequenced. Interaction sequence tags were analyzed by multiparallel BLAST analysis.

**Indirect immunofluorescence assays**

For the images shown in Figure 4B, Hoechst-stained DNA was detected using FS01 modified reflector (excitation wavelength 359-371 nm; dichroic glass FT 395; emission wavelength 421-479 nm); PB2-GFP-comp was detected using FS44 (Zeiss) reflector (excitation wavelength 455-495 nm; dichroic glass FT 500; emission wavelength 505-555 nm) ; DyLight 633-stained RED was detected using XF110-2 (Omega) reflector (excitation wavelength 604-645 nm; dichroic glass FT 650; emission wavelength 668-723 nm) ; DyLight 550-stained SMU1 was detected using XF37 (Omega) reflector (excitation wavelength 540-552 nm; dichroic glass FT 550; emission wavelength 565-595 nm).

| **Image** | **DNA (Hoechst)** | **PB2 (GFP)** | **RED (DL633)** | **SMU1 (DL550)** | **Resolution** | **Bit depth** | **Binning** | **Lens** |
| --- | --- | --- | --- | --- | --- | --- | --- | --- |
| Mock | 3ms | 600 ms | 1300ms | 70ms | 1388x1040 | 16bits | Std mono | X40 |
| WSN | 3ms | 600 ms | 1300ms | 70ms | 1388x1040 | 16bits | Std mono | X40 |

For the images shown in Figure 5, Hoechst-stained DNA was detected using FS01 modified reflector (excitation wavelength 359-371 nm; dichroic glass FT 395; emission wavelength 421-479 nm); PB2-GFP-comp was detected using FS44 (Zeiss) reflector (excitation wavelength 455-495 nm; dichroic glass FT 500; emission wavelength 505-555 nm) ; Alexa fluor 555-stained IK was detected using XF37 (Omega) reflector (excitation wavelength 540-552 nm; dichroic glass FT 550; emission wavelength 565-595 nm).

| **Image** | **DNA (Hoechst)** | **PB2 (GFP)** | **mCherry** | **Resolution** | **Bit depth** | **Binning** | **Lens** |
| --- | --- | --- | --- | --- | --- | --- | --- |
| Control  PB2-GFP11 | 185ms | 392ms | 6ms | 1388x1040 | 16bits | Std mono | X63 |
| mCherry-IK  PB2-GFP11 (left) | 185ms | 392ms | 6ms | 1388x1040 | 16bits | Std mono | X63 |
| mCherry-IK  PB2-GFP11 (right) | 185ms | 392ms | 6ms | 1388x1040 | 16bits | Std mono | X63 |
| mCherry-IK  PB2-wt | 185ms | 392ms | 6ms | 1388x1040 | 16bits | Std mono | X63 |
| mCherry  PB2-wt | 185ms | 392ms | 6ms | 1388x1040 | 16bits | Std mono | X63 |

For the images shown in Figure 9, Hoechst-stained DNA was detected using FS01 modified reflector (excitation wavelength 359-371 nm; dichroic glass FT 395; emission wavelength 421-479 nm); Alexa fluor 488 stained NP was detected using FS44 (Zeiss) reflector (excitation wavelength 455-495 nm; dichroic glass FT 500; emission wavelength 505-555 nm).

| **Image** | **DNA (Hoechst)** | **NP (AF488)** | **Resolution** | **Bit depth** | **Binning** | **Lens** |
| --- | --- | --- | --- | --- | --- | --- |
| NT siRNA | 5ms | 80ms | 1388x1040 | 16bits | Std mono | X40 |
| RED siRNA | 5ms | 80ms | 1388x1040 | 16bits | Std mono | X40 |

For the images shown in Supplementary Figure S2, Hoechst-stained DNA was detected using FS01 modified reflector (excitation wavelength 359-371 nm; dichroic glass FT 395; emission wavelength 421-479 nm); AF555-stained RED was detected using XF37 (Omega) reflector (excitation wavelength 540-552 nm; dichroic glass FT 550; emission wavelength 565-595 nm); DyLight 550-stained SMU1 was detected using XF37 (Omega) reflector (excitation wavelength 540-552 nm; dichroic glass FT 550; emission wavelength 565-595 nm).

| **Image** | **DNA (Hoechst)** | **RED (AF555)** | **SMU1 (DL550)** | **Resolution** | **Bit depth** | **Binning** | **Lens** |
| --- | --- | --- | --- | --- | --- | --- | --- |
| NT siRNA | 3ms | 136ms | - | 1388x1040 | 16bits | Std mono | X40 |
| RED siRNA | 3ms | 136ms | - | 1388x1040 | 16bits | Std mono | X40 |
| NT siRNA | 4ms | - | 150ms | 1388x1040 | 16bits | Std mono | X40 |
| RED siRNA | 4ms | - | 150ms | 1388x1040 | 16bits | Std mono | X40 |

For the images shown in Supplementary Figure S4, Hoechst-stained DNA was detected using FS01 modified reflector (excitation wavelength 359-371 nm; dichroic glass FT 395; emission wavelength 421-479 nm); Alexa fluor 488 stained NP was detected using FS44 (Zeiss) reflector (excitation wavelength 455-495 nm; dichroic glass FT 500; emission wavelength 505-555 nm).

| **Image** | **DNA (Hoechst)** | **NP (AF488)** | **Resolution** | **Bit depth** | **Binning** | **Lens** |
| --- | --- | --- | --- | --- | --- | --- |
| NT siRNA | 107ms | 200ms | 1388x1040 | 16bits | Std mono | X40 |
| RED siRNA | 164ms | 200ms | 1388x1040 | 16bits | Std mono | X40 |

**Reverse transcription - quantitative PCR assays**

The sequence of the primers and probes used for RT-qPCR analysis of viral mRNAs are indicated in the table below, as well as the software used for design, the melting temperature (Tm) and size in nucleotide (nt), and modifications applied to the Taqman probes. for each oligonucleotide. The size of the expected amplification products, in base pairs (bp), is also indicated.

| **Primer** | **Sequence** | **Software** | **Size** | **Tm** | **Modification** | **Product** |
| --- | --- | --- | --- | --- | --- | --- |
| M1TaqFw | 5’-TTCTCTCTATCGTCCC-3’ | CLC | 16nt | 48,5°C |  | 156bp |
| M1TaqProbeS | 5‘-ACCAATCCTGTCACCT-3’ | CLC | 16nt | 54,6°C | 5’-FAM, 3’BHQ1 |  |
| M1TaqRv | 5’-CAAATCCTAAAATCCCC-3’ | CLC | 17nt | 48,7°C |  |  |
| M2TaqFw | 5’-GCAAAAGCAGGTAGATATTGA-3’ | Primer3 | 21nt | 54,8°C |  | 108bp |
| M2TaqProbeS | 5’-AGGTCGAAACGCCTATCAGAAAC-3’ | Primer3 | 23nt | 62,9°C | 5’-FAM, 3’BHQ1 |  |
| M2TaqRv | 5’-AATGACGAGAGGATCACTTG-3’ | Primer3 | 20nt | 55,2°C |  |  |
| NS1TaqFw | 5’-CTTTCTTTGGCATGTC-3’ | CLC | 16nt | 49,3°C |  | 161bp |
| NS1TaqProbeAS | 5’-TGCTGCCTCTTCCTCTT-3’ | CLC | 17nt | 56,2°C | 5’-FAM, 3’BHQ1 |  |
| NS1TaqRv | 5’-GCTCCACTATTTGCTT -3’ | CLC | 16nt | 49,8°C |  |  |
| NS2TaqFw | 5’-GGGTGACAAAGACATAATGG-3’ | Primer3 | 20nt | 55,3°C |  | 177bp |
| NS2TaqProbeS | 5’-CAAGCTTTCAGGACATACTGATGA-3’ | Primer3 | 24nt | 60,7°C | 5’-FAM, 3’BHQ1 |  |
| NS2TaqRv | 5’-TCTCCCATTCTCATTACTGC-3’ | Primer3 | 20nt | 55,3°C |  |  |
