# Supplementary material for: Correction: Recruitment of RED-SMU1 Complex by Influenza A Virus RNA Polymerase to Control Ciral mRNA Splicing
Source: PLoS Pathog. 2014 Jul 23;10(7):e1004317. doi: 10.1371/journal.ppat.1004317 (PMC4108404; doi:10.1371/journal.ppat.1004317)
Supplement: File S2 — Republished, corrected article (PDF) [file ppat.1004317.s002.pdf]

OPEN ACCESS Freely available online

PLOS | PATHOGENS

Recruitment of RED-SMU1 Complex by Influenza A Virus RNA Polymerase to Control Viral mRNA Splicing

Guillaume Fournier[1,2,3]⁹, Chiayn Chiang[1,2,3]⁹¤a, Sandie Munier[1,2,3], Andru Tomoiu[1,2,3]¤b, Caroline Demeret[1,2,3], Pierre-Olivier Vidalain[2,4], Yves Jacob[1,2,3], Nadia Naffakh[1,2,3]*

1 Institut Pasteur, Unité de Génétique Moléculaire des Virus à ARN, Département de Virologie, Paris, France, **2** CNRS, UMR 3569, Paris, France, **3** Université Paris Diderot, Sorbonne Paris Cité, Unité de Génétique Moléculaire des Virus à ARN, Paris, France, **4** Institut Pasteur, Unité de Génomique Virale et Vaccination, Département de Virologie, Paris, France

Abstract

Influenza A viruses are major pathogens in humans and in animals, whose genome consists of eight single-stranded RNA segments of negative polarity. Viral mRNAs are synthesized by the viral RNA-dependent RNA polymerase in the nucleus of infected cells, in close association with the cellular transcriptional machinery. Two proteins essential for viral multiplication, the exportin NS2/NEP and the ion channel protein M2, are produced by splicing of the NS1 and M1 mRNAs, respectively. Here we identify two human spliceosomal factors, RED and SMU1, that control the expression of NS2/NEP and are required for efficient viral multiplication. We provide several lines of evidence that in infected cells, the hetero-trimeric viral polymerase recruits a complex formed by RED and SMU1 through interaction with its PB2 and PB1 subunits. We demonstrate that the splicing of the NS1 viral mRNA is specifically affected in cells depleted of RED or SMU1, leading to a decreased production of the spliced mRNA species NS2, and to a reduced NS2/NS1 protein ratio. In agreement with the exportin function of NS2, these defects impair the transport of newly synthesized viral ribonucleoproteins from the nucleus to the cytoplasm, and strongly reduce the production of infectious influenza virions. Overall, our results unravel a new mechanism of viral subversion of the cellular splicing machinery, by establishing that the human splicing factors RED and SMU1 act jointly as key regulators of influenza virus gene expression. In addition, our data point to a central role of the viral RNA polymerase in coupling transcription and alternative splicing of the viral mRNAs.

Citation: Fournier G, Chiang C, Munier S, Tomoiu A, Demeret C, et al. (2014) Recruitment of RED-SMU1 Complex by Influenza A Virus RNA Polymerase to Control Viral mRNA Splicing. PLoS Pathog 10(6): e1004164. doi:10.1371/journal.ppat.1004164

Editor: Andrew Pekosz, Johns Hopkins University - Bloomberg School of Public Health, United States of America

Received January 23, 2014; **Accepted** April 21, 2014; **Published** June 12, 2014

Copyright: © 2014 Fournier et al. This is an open-access article distributed under the terms of the Creative Commons Attribution License, which permits unrestricted use, distribution, and reproduction in any medium, provided the original author and source are credited.

Funding: This work was funded by the European Commission funded FP7 project FLUPHARM, Grant Agreement No 259751 (http://flupharm.eu). The funders had no role in study design, data collection and analysis, decision to publish, or preparation of the manuscript.

Competing Interests: The authors have declared that no competing interests exist.

* E-mail: nadia.naffakh@pasteur.fr

⁹ These authors contributed equally to this work.

¤a Current address: TaiGen Biotechnology Co. Ltd., Taipei, Taiwan
¤b Current address: Université Laval, Quebec, Quebec, Canada

Introduction

Viruses are dependent on host cell functions for their replication. Unlike most viruses with an RNA genome, influenza A viruses replicate in the nucleus of infected cells and some of the viral RNAs undergo splicing. Their genome consists of eight single-stranded RNA segments of negative polarity encoding ten major proteins and several auxiliary proteins. Each viral RNA (vRNA) segment is encapsidated with the nucleoprotein (NP) and associated with the heterotrimeric viral RNA-dependent RNA polymerase consisting of the PB1, PB2 and PA subunits, to form a viral ribonucleoprotein complex (vRNP). After viral entry by endocytosis, incoming vRNPs are released into the cytoplasm and then imported into the nucleus. The viral polymerase ensures the transcription of vRNAs into mRNAs, and their replication via the synthesis of full-length complementary RNAs (cRNAs) which in turn serve as templates for the synthesis of vRNAs.

Although viral mRNAs harbour the 5′ cap and 3′ poly(A) tail structures characteristic of cellular mRNAs, their synthesis proceeds through very distinct mechanisms (for a review, see [1,2]). The initiation of transcription involves a cap-snatching mechanism by which the PB2 subunit of the viral polymerase binds to the 5′ cap of cellular pre-mRNAs, the PA subunit ensures a cleavage 10–15 nucleotides downstream the cap, and the resulting short capped oligonucleotide is used by the PB1 subunit as a primer for elongation, using a vRNA as a template. Termination and polyadenylation occur at a stretch of five to seven U residues close to the 5′ end of the template, which is reiteratively copied by the viral polymerase. The stuttering of the viral polymerase is likely due to the fact that it remains bound to the 5′ end of the vRNA template during elongation, and thus encounters steric hindrance when having transcribed most of the template in the 3′→5′ direction.

Unlike cellular pre-mRNAs, most viral mRNAs are intron-less. However, the two smallest vRNA segments in size, M and NS, produce mRNAs that undergo splicing. The M segment gives rise to the unspliced M1 and spliced M2 mRNAs, which encode the M1 matrix protein and M2 ion channel protein, respectively. Alternatively spliced transcripts of segment M encode a small polypeptide of unknown function [3], and for some viral strains the

Author Summary

Influenza A viruses are major pathogens which pose continuous animal and public health challenges. Enhancing the knowledge of their life cycle, and especially the understanding of how viral components interact with the host cell, is essential to achieve better prevention and treatment of the disease. The polymerase of influenza A viruses plays a central role in the viral cycle, notably by driving the synthesis of viral messenger RNAs that are translated into viral proteins. Here we identify two human splicing factors, RED and SMU1, that associate with the viral polymerase. We show that these factors jointly regulate the splicing of the NS1 messenger RNA into the shorter NS2 messenger RNA, which encodes a key viral protein named NS2/NEP. We demonstrate that RED and SMU1 are required for efficient expression of NS2/NEP, and for the NS2/NEP-mediated intracellular trafficking of viral components. Overall, our results show that RED and SMU1 are essential for the replication of influenza A viruses. With respect to the need of novel anti-influenza therapies for epidemic and pandemic preparedness, the functional and physical interactions between these cellular splicing factors and the viral transcriptional machinery could be targeted to inhibit viral replication.

M42 variant of the M2 ion channel [4]. The NS segment gives rise to the unspliced NS1 and spliced NS2 mRNAs, which encode two multifunctional proteins: the non-structural NS1 protein, an interferon antagonist which counters cellular antiviral responses (reviewed in [5]), and the NS2/NEP protein which is involved both in viral genome replication and nuclear export of neo-synthetized vRNPs (reviewed in [6]). An alternatively spliced transcript of segment NS, encoding a truncated version of NS1 named NS3, has been reported for some viral strains [7].

The splicing of viral pre-mRNAs is highly controlled so that a majority of unspliced transcripts and a minority of spliced transcripts are produced, unlike the splicing of cellular pre-mRNAs which is generally very efficient. Previous studies, mainly focused on M1 mRNA, identified some cis-acting RNA signals, viral and host proteins involved in viral mRNA splicing regulation. Mutations at alternative 5′ splice sites of M1 mRNA [8] or NS1 mRNA [9] negatively affect viral growth rates. A secondary structure in the NS1 mRNA intron was detected, but was not functionally investigated [10]. Alternative splicing of M1 mRNA is controlled by the viral polymerase [11] and the viral NS1 protein [12]. Whether or not the NS1 protein controls NS1 mRNA splicing remains controversial [13,14]. Influenza virus dependency on the cellular splicing machinery is highlighted by a series of genome-wide RNAi screens, aimed at identifying cellular proteins that are required for influenza virus replication. The different screens show little overlap when analyzed at the level of individual proteins, but a much higher degree of overlap when analyzed at the level of cellular functions and pathways (reviewed in [15,16]). Splicing/processing of pre-mRNAs is one of the most overrepresented gene ontology (GO) category, together with intracellular transport and kinase-mediated signaling [15]. Whereas a few cellular proteins were shown to be involved in splicing regulation of the M1 viral mRNA, *i.e.* the cellular SF2/ASF splicing factor [17], the hnRNP K, and the NS1-BP proteins [18], little is known about regulation of NS1 mRNA splicing by cellular factors.

In this study, we identified the RED spliceosomal factor as an interacting partner of influenza A virus polymerase. The homologues of RED in *Caenorhabditis elegans* and *Arabidopsis thaliana* were shown to work cooperatively with SMU1 to regulate the splicing of specific pre-mRNAs [19,20,21]. We thus investigated the role of RED and SMU1 in influenza A virus replication. We provide several lines of evidence that RED mediates the association of RED-SMU1 complexes with the viral RNA polymerase. We analyzed the effects of RED or SMU1 depletion on the splicing efficiency of viral mRNAs, the production of viral proteins, and the release of infectious viral particles. Overall, our results show that RED and SMU1 both promote influenza virus replication, by jointly controlling splicing of the viral NS1 mRNA and production of the essential NS2/NEP protein.

Results

Identification of RED as an interactor of influenza virus polymerase in a yeast two-hybrid screen

Structural data indicate that the PB1, PB2 and PA subunits of influenza virus polymerase are tightly associated [22,23], suggesting that some interactions of the viral polymerase with host factors are either mediated by more than one viral polymerase subunit, or dependent on conformational transition upon polymerase complex formation. We thus developed an original yeast two-hybrid system where two viral polymerase subunits were co-expressed in yeast to be used as bait. Only one viral polymerase subunit was fused to Gal4 DNA binding domain (DB), whereas the other one was co-expressed in its native form and addressed to the yeast nucleus. Four distinct combinations, namely [DB-PB1+PA], [PB1+DB-PA], [DB-PB1+PB2], and [PB1+DB-PB2], were used as baits to screen human cDNA libraries from spleen and foetal brain (see Methods S1 for details on the procedure). Numerous hits were obtained with the PB1-PA dimer, but only few with the PB1-PB2 dimer. This observation suggests that the PB1-PB2 bait is not suitable for yeast two-hybrid, possibly because it prevents efficient reconstitution of a functional Gal4 protein. Altogether, 37 high-confidence interactors (≥3 hits) and 164 low-confidence interactors (1 or 2 hits) of the viral polymerase were identified (Supplemental Table S1). The RED/IK/RER protein appeared as a predominant interactor of the PB1-PA dimer in the high-confidence group with 184 hits, and had not been identified in previous yeast two-hybrid screens (highlighted in the Supplemental Table S1). RED, which is 557 amino acid long and owes its name to the presence of a repetitive stretch of arginine (R), glutamic acid (E) and aspartic acid (D) residues from position 334 to 375 [24], was identified in two independent studies as a component of the spliceosome [25,26].

Interaction of RED with individual subunits of the viral polymerase

We next characterized the ability of RED to interact with each individual subunit of the viral polymerase in human cells. To this end, we used a split-luciferase based trans-complementation assay as described earlier [27]. Briefly, an expression plasmid encoding the RED protein fused to the Gluc2 fragment of *Gaussia princeps* luciferase was produced. HEK-293T cells were transfected with the Gluc2-RED expression plasmid, together with plasmids that allowed the expression of PB1, PB2 or PA polymerase subunit fused to the trans-complementing *Gaussia princeps* luciferase Gluc1 fragment. Additional cellular proteins were used as positive controls: IPO5, known to interact with the PB1 protein and the PB1-PA dimer but not with the PB2 protein [28]; KPNA2 and hCLE, known to interact with PB2 and PA, respectively [29,30]; and SMU1, found as an interactor of RED in yeast two-hybrid and co-immunoprecipitation assays [31]. Luciferase activity measured in cell extracts was expressed as a Normalized

Luminescence Ratio (NLR) over control protein pairs (Figure 1A). An NLR cut-off of 8 discriminated interacting pairs from non-interacting pairs with a false positive background below 2.5%, as determined with a random set of human proteins (see [27] and Materials and Methods for details on the procedure). Luciferase activities detected in the presence of Gluc2-IPO5, -KPNA2 and -hCLE proteins were in agreement with the interaction patterns described in the literature (Figure 1B, grey bars). In the same conditions, binary RED-PB2 and RED-PB1 interactions, but no RED-PA interaction, were detected (Figure 1B, black bars). These observations suggest that the RED protein makes contact with a polymerase region overlapping PB1 and PB2, and that interaction of RED with the [DB-PB1+PA] and [PB1+DB-PA] dimers in yeast was mediated by the RED-PB1 interaction. The fact that RED-PB2 interaction was not detected in yeast probably comes from a general unsuitability of the PB1-PB2 bait for yeast two-hybrid, as mentioned above. The high levels of luciferase activity measured in the presence of Gluc2-RED and Gluc1-SMU1 demonstrated that a strong RED-SMU1 interaction occurs in cultured human cells (Figure 1B, hatched bar); using the same assay, no binary interaction between SMU1 and any of the three subunits of the viral polymerase was detected (Figure 1B, open bars).

Copurification of RED with the viral polymerase from infected cell extracts

To further document the association of RED with the viral polymerase in infected cells, we used a recombinant influenza A virus (WSN strain) encoding a PB2 protein fused to the Strep tag at its C-terminal end (rWSN-PB2-Strep), which grows as efficiently as its untagged counterpart (rWSN) [32,33]. Lysates were prepared from HEK-293T cells infected with rWSN-PB2-Strep or rWSN, and then subjected to Strep purification using StrepTactin as previously described [32,33]. This protocol allows co-purification of PB2-Strep with similar amounts of PB1 and PA and with larger amounts of NP protein [32,33], which most likely correspond to a mixture of vRNPs and free trimeric polymerase although the presence of limited amounts of cRNPs and isolated PB2-Strep protein cannot be excluded. Western blot analysis showed that the cellular RED protein was specifically co-purified with PB2-Strep (Figure 1C, middle panel). Remarkably, RED's cellular partner SMU1 was also specifically co-purified with vRNPs (Figure 1C, lower panel). The two bands detected with the anti-SMU1 antibody, in the lysates as well as in the eluates, most likely correspond to two distinct forms of the SMU1 protein as they are both sensitive to treatment with anti-SMU1 siRNAs (as shown below). The recovery yield was lower for RED and SMU1 than for PB2-Strep. The co-purification experiment was repeated with a natural human influenza virus isolate. To this end, we used the recombinant P908-WSN and P908-Cstrep-WSN viruses, whose PB1, PB2 or PB2-Strep, PA and NP genes derive from the A/Paris/908/97 (P908) virus [34]. Again, the RED and SMU1 proteins were specifically co-purified with PB2-Strep (Supplemental Figure S1A).

Mapping of the interaction domain of RED with the viral polymerase

We asked which domain(s) of RED were able to bind the monomeric PB1 and PB2 subunits and the SMU1 protein, using the *Gaussia princeps* luciferase-based complementation assay. To

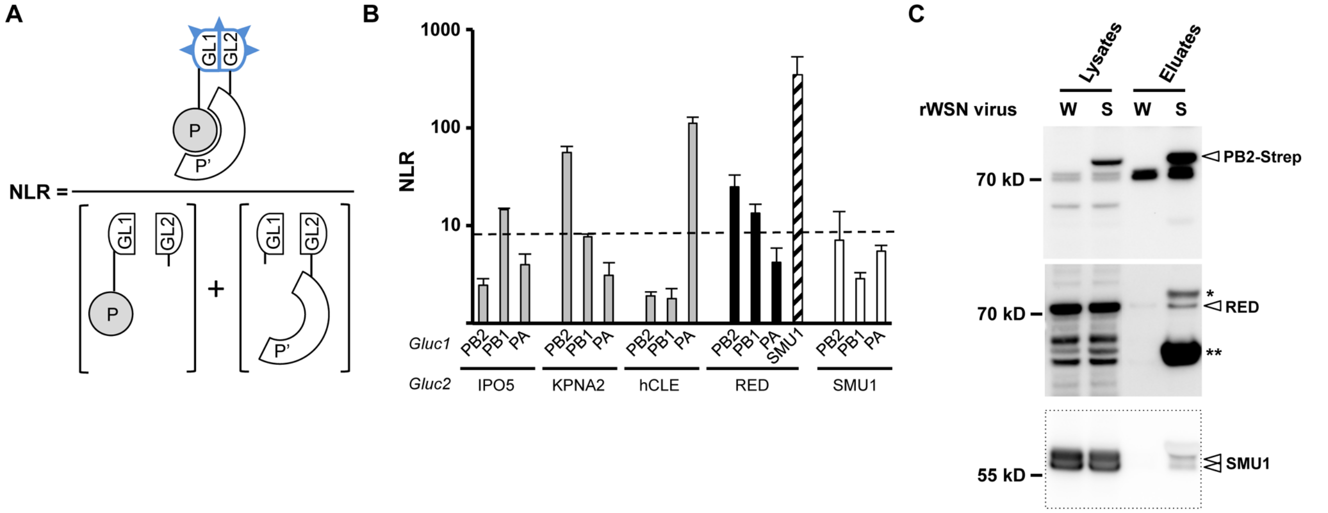

Figure 1. Interaction of RED with influenza virus polymerase in human cells. A. Schematic representation of the *Gaussia princeps* luciferase-based complementation assay and Normalized Luminescence Ratios (NLRs) calculation method. The luminescence activity measured in cells co-transfected with the plasmids encoding the P-Gluc1 and P′-Gluc2 fusion proteins is divided by the sum of the luminescence activities measured in control samples co-transfected with either the P-Gluc1 and Gluc2 plasmids or the Gluc1 and P′-Gluc2 plasmids. For details, see the Materials and Methods section, and [27]. **B**. For each indicated pair of Gluc1 and Gluc2 fusion proteins, the NLR was determined as described in A. The data are expressed as the mean +/− SD of triplicates and are representative of two independent experiments. The dashed line indicates the NLR cut-off value that reduces false positive background below 2.5%, as determined using a random reference set of human proteins. **C**. Co-purification of the endogenous RED and SMU1 proteins with the viral polymerase in infected cells. HEK-293T cells were infected at a m.o.i. of 5 with recombinant wild-type rWSN (W) or rWSN-PB2-Strep (S) viruses and incubated at 37°C for 6 hours. Whole-cell lysates were prepared and a fraction was incubated with StrepTactin beads as described in Material and Methods. Protein complexes were eluted, loaded on a 4–12% SDS-polyacrylamide gel and analyzed by western blotting using either StrepTactin to detect the PB2-Strep protein (upper panel) or an antibody specific for the RED or SMU1 protein (middle and lower panels). * and **: non-specific detection of the PB2 and NP protein, respectively, as inferred from previous experiments [33]. The bands detected in the PB2-Strep panel at 70 kD and the faster migrating band (present only in lysates) were also detected in mock-infected cells (data not shown).

doi:10.1371/journal.ppat.1004164.g001

this end, we produced expression plasmids encoding a Gluc2-tagged N-terminal domain of RED (Nt-RED, residues 1 to 315), or C-terminal domain of RED (Ct-RED, residues 316 to 557). The delimitation of Nt-RED and Ct-RED was based on the fact that a secretory form of RED starting at residue 316 is naturally produced in hematopoietic cells [35,36] (Figure 2A). Plasmids encoding a Gluc2-tagged RED, Nt-RED or Ct-RED were co-transfected with the PB2-Gluc1, PB1-Gluc1 or Gluc1-SMU1 plasmids. For each protein combination, luciferase activity was expressed as a NLR over control protein pairs. The Nt-RED domain bound as efficiently as the full-length RED protein to PB1 and SMU1, but less efficiently to PB2 as indicated by a 75% reduction of the corresponding NLR (Figure 2B). The Ct-RED domain did not bind efficiently to PB1 and PB2 nor to SMU1 (Figure 2B). Low NLR values observed in the presence of the Gluc2-tagged Ct-RED domain were not due to defective expression, as shown by western-blot analysis (Figure 2C). Overall, our data indicate that the N-terminal domain of RED is mediating RED's interaction with both the viral polymerase PB1 and PB2 subunits and with SMU1, whereas its C-terminal domain enhances specifically the interaction with PB2.

To document the interaction domain of RED with the viral polymerase in an infected cell context, we produced expression plasmids encoding an HA-tagged full-length RED, Nt-RED or Ct-RED. HEK-293T cells were transfected with these plasmids and infected with the rWSN or rWSN-PB2-Strep virus. Strep purification followed by western blot analysis using an anti-HA antibody revealed that, unlike the Ct-RED domain, the Nt-RED domain interacted specifically with PB2-Strep (Figure 2D), in agreement with the protein-protein interaction assay shown in Figure 2B. The same experiment performed with the recombinant P908/WSN and P908-Cstrep-WSN viruses gave similar results (Supplemental Figure S1B). Therefore, in cells infected with two distinct influenza A viruses, RED appears to associate with PB2-Strep, most likely when incorporated into polymerase complexes or vRNPs, through its N-terminal domain.

Association of RED-SMU1 complexes with the viral polymerase

Based on our findings that i) the human RED and SMU1 proteins co-purify with influenza virus polymerase, ii) RED and SMU1 interact strongly with each other, and ii) RED but not SMU1 interacts with influenza virus polymerase PB2 and PB1 subunits, we hypothesized that RED was mediating the association of RED-SMU1 complexes with influenza virus polymerase. In order to test this hypothesis, the protein complementation assay using PB2-Gluc1 and Gluc2-SMU1 plasmids was repeated in the absence or in the presence of over-expressed RED protein, and NLR were determined. Overexpression of the RED protein resulted in a 6-fold increase of the NLR, bringing it above the NLR cut-off value for non-interacting pairs (two independent experiments in triplicate, two-way ANOVA, unweighted means, $p<0.001$) (Figure 3A). These data suggested that, although no direct SMU1-PB2 interaction occurs, an indirect association of SMU1 and PB2 can be mediated by the formation of ternary PB2-RED-SMU1 complexes.

To further document the existence of such ternary complexes, we examined whether [Gluc1-SMU1+Gluc2-RED] complexes could be co-purified with PB2. The PB2 protein of WSN virus, either wild-type or fused to the Strep tag, was co-expressed in HEK-293T cells together with [Gluc1-SMU1+Gluc2-RED], or with the [Gluc1+Gluc2-RED] and [Gluc1-SMU1+Gluc2] control pairs. Alternatively, the rWSN-PB2-Strep virus or the control rWSN virus were used to infect HEK-293T cells that expressed [Gluc1-SMU1+Gluc2-RED], [Gluc1+Gluc2-RED] or [Gluc1-SMU1+Gluc2]. Lysates were prepared at 24 hours post-transfection or 6 hours post-infection and subjected to Strep purification using StrepTactin beads. The NLRs measured in the lysates were similar whether PB2 or PB2-Strep was expressed (Figure 3B, upper graph). In contrast, NLRs associated with StrepTactin were about 8-fold and 3-fold higher in the PB2-Strep samples than in the control PB2 samples, in the transfection and infection setting, respectively (Figure 3B, lower graph). Overall, our data indicate that PB2-RED-SMU1 complexes are being formed in influenza virus infected cells.

Accumulation and localization of RED and SMU1 in infected cells

The steady-state level and subcellular localization of endogenous proteins RED and SMU1 were monitored throughout the viral cycle. A549 cells were mock-infected or infected with the WSN virus at a high multiplicity of infection (m.o.i.). Total cell extracts were prepared at various times post-infection and were analyzed by western blot using anti-RED, anti-SMU1 and anti-NP antibodies. As shown in Figure 4A, RED and SMU1 intracellular levels did not undergo major variations during influenza virus infection. When analysed by indirect immunofluorescence, the RED and SMU1 proteins (pseudo-colored in red and cyan, respectively, in Figure 4B) were both localized in the nucleus and excluded from the nucleoli, as previously reported for RED [37]. The specificity of these immunostainings was confirmed by the fact that RED and SMU1 signals were strongly reduced in siRNA-treated cells (Supplemental Figure S2). The intensity and subcellular distribution of RED and SMU1 were unchanged in infected cells compared to control cells, when observed at 6 hpi (Figure 4B).

We then used the rWSN-PB2-GFP11 virus [38] to infect HEK-293T cells that transiently expressed GFP1-10 and an mCherry-RED fusion protein. The over-expressed mCherry-RED protein appeared concentrated within large intra-nuclear dots (Supplemental Figure S3, pseudo-colored in red), a subcellular localisation very distinct from the diffuse intranuclear localisation observed for the endogenous RED protein (Figure 4B). A clear re-localisation of PB2-GFP_{comp} into the mCherry-RED dots was observed upon infection (Supplemental Figure S3, pseudo-colored in green, and merge), further supporting a specific RED-PB2 interaction in influenza-virus infected cells. This phenomenon was not observed when a control mCherry protein was expressed (Supplemental Figure S3, right row).

Cross effects of RED and SMU1 knock-down on influenza virus replication

To assess the functional contribution of the RED and SMU1 proteins to the viral life cycle, we performed siRNA-mediated knock-down in A549 cells. A pool of four RED siRNAs (Rp) and two individual RED siRNAs (R1 and R2) were used, together with a pool of four SMU1 siRNAs (Sp) and control non-target siRNAs (NT). Cell lysates were analyzed for the presence of RED and SMU1 by western blotting. The steady-state levels of RED showed a 90% reduction 36 hours after RED siRNA treatment; however, RED was silenced almost as efficiently by SMU1 siRNAs (Figure 5A, left panel). Conversely, SMU1 appeared to be silenced by RED siRNAs Rp, R1 or R2 nearly as well as by SMU1 siRNAs (Figure 5A, right panel). A cell viability assay was performed in parallel. Little or no reduction of the cell viability signal was measured in RED or SMU1 silenced cells compared to control siRNA-treated cells (Figure 5B). Our findings were in agreement

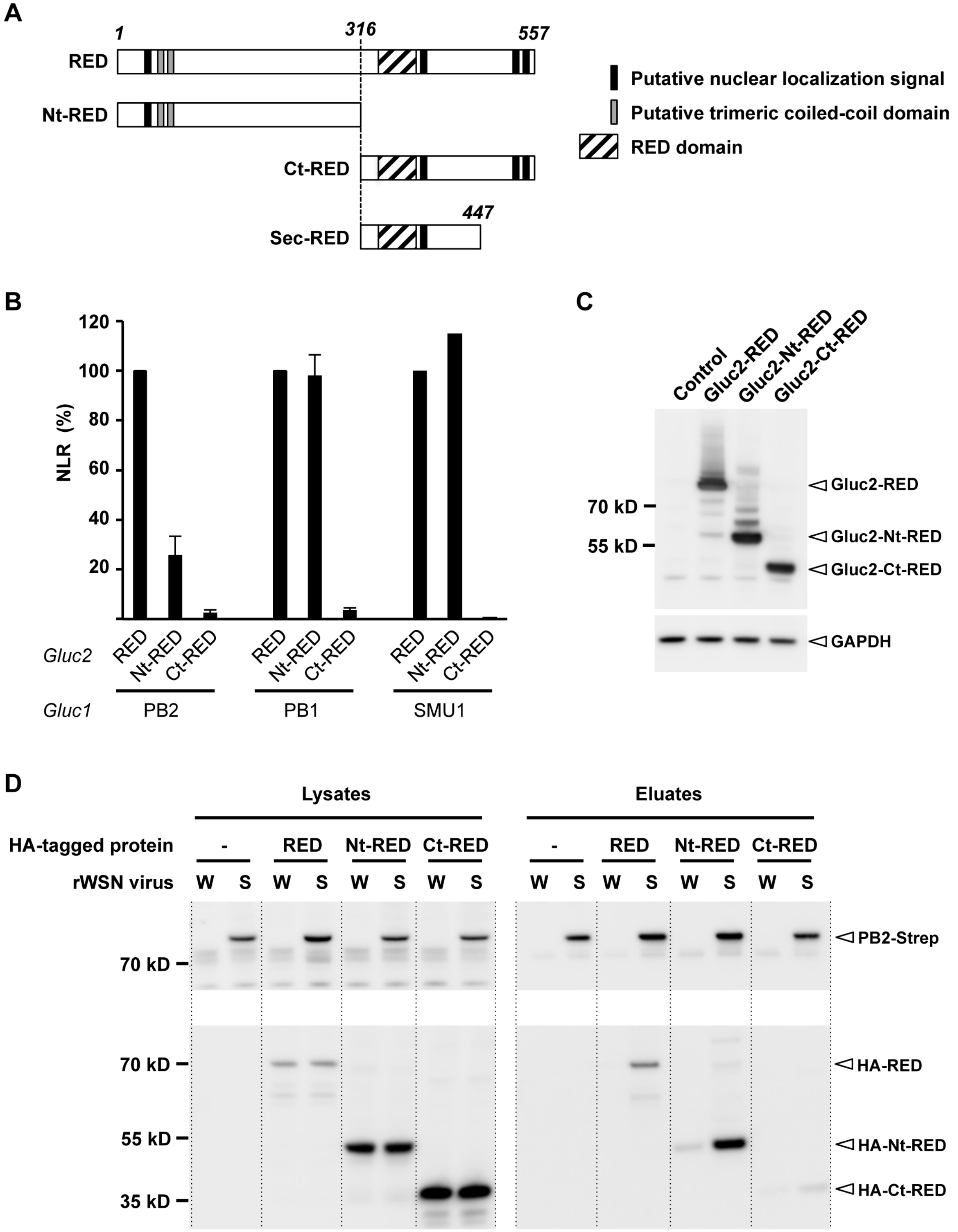

Figure 2. Mapping of the interaction domain of RED with the viral polymerase. A. Schematic representation of the full-length RED, N-terminal domain (Nt-RED) and C-terminal domain (Ct-RED) of RED that were transiently expressed in the experiment shown in B. The domain corresponding to the truncated, secreted form of RED (sec-RED) is also represented. **B**. For each indicated pair of Gluc1 and Gluc2 fusion proteins, the NLR was determined as described in Figure 1A. The NLR are expressed as percentages (100%: the NLR measured in the presence of Gluc2-RED). The data are expressed as the mean +/− SD and are representative of two independent experiments (PB1, PB2) or a single experiment (SMU1) in triplicates. **C**. Steady-state levels of the recombinant Gluc2-RED, Gluc2-Nt-RED and Gluc2-Ct-RED proteins. HEK-293T cells were transfected with the Gluc2-RED, Gluc2-Nt-RED, Gluc2-Ct-RED plasmids, or mock-transfected with the empty pCI plasmid (control). Total cell extracts were prepared 24 hours post-transfection, loaded on a 4–12% SDS-polyacrylamide gel and analyzed by western blotting using a polyclonal antibody specific for

Gaussia princeps luciferase. **D**. Co-purification of the viral polymerase and recombinant, HA-tagged polypeptides corresponding to full-length or truncated forms of the RED protein. HEK-293T cells were transfected with the HA-RED, HA-Nt-RED or HA-Ct-RED expression plasmids, or mock-transfected with the pCI plasmid (−). 24 hours post-transfection, they were infected at a m.o.i. of 5 with recombinant WSN (W) or WSN-PB2-Strep (S) viruses and incubated at 37°C for 6 hours. Whole-cell lysates were prepared and a fraction was incubated with StrepTactin beads as described in Material and Methods. Protein complexes were eluted, loaded on a 4–12% SDS-polyacrylamide gel and analyzed by western blotting using either StrepTactin to detect the PB2-Strep protein (upper panel) or a monoclonal antibody specific for the HA tag (lower panel).
doi:10.1371/journal.ppat.1004164.g002

with published data on cross effects of SMU1 and SMU2 gene inactivation in *Caenorhabditis elegans* [19]. Taken together with the fact that RT-qPCR analysis revealed no reduction of SMU1 mRNA levels upon RED siRNA treatment, and no reduction of RED mRNA levels upon SMU1 siRNA treatment (Supplemental Figure S4), our observations strongly suggest that human RED and SMU1 proteins are stabilizing each other within the cell.

The effect of RED or SMU1 knock-down on WSN influenza virus replication was then examined. A549 cells treated with RED, SMU1, or control siRNAs were infected at a m.o.i. of 0.001 pfu/cell with the WSN influenza virus, and the production of infectious viral particles was evaluated at 24 and 48 hpi. Viral titers in supernatants of RED- and SMU1-silenced cells showed a similar 2-log reduction at 24 hpi and a 2 to 3-log reduction at 48 hpi compared to control cells (two independent experiments in triplicate, two-way ANOVA on logarithmic-transformed data, unweighted means, $p<0.0001$) (Figure 5C, circle symbols). No such significant differences were observed upon infection with VSV Indiana strain, a negative-stranded RNA virus which replicates in the cytoplasm (Figure 5C, triangle symbols), nor with Adenovirus 5 which replicates in the nucleus and is dependent on the cellular splicing machinery (Figure 5C, square symbols). Individual anti-RED siRNAs R1 and R2 showed the same effect as the Rp siRNA pool, *i.e.* they strongly inhibited the production of influenza infectious particles without affecting the production of VSV infectious particles (Figure 5D). Overall our data show that RED and SMU1 are specifically required for efficient influenza virus multiplication. However, as RED and SMU1 proteins are stabilizing each other, these knock-down experiments do not allow to discern whether RED, SMU1 or both are playing an active role in influenza virus multiplication.

Knock-down of RED or SMU1 reduces the NS2/NS1 mRNA and protein ratios in infected cells

We hypothesized that the viral replication defect in RED- and SMU1-depleted cells could be due to an impaired splicing of viral mRNAs. We thus examined the effect of RED or SMU1 depletion on the accumulation of viral mRNAs encoding the NS1, NS2, M1 and M2 proteins. A549 cells treated with RED, SMU1 or control NT siRNAs were infected with influenza virus WSN at a m.o.i. of 5 pfu/cell. Poly-adenylated RNAs were purified and RT-qPCR was performed using specific primers and probes as described in Figure 6A. The mRNA copy numbers, shown in Figure 6B, were determined using the standard curve method and normalized with respect to GAPDH mRNA copy numbers (see the Materials and Methods section for details). In agreement with previously published data [13,18,39], mRNA levels showed a sharp increase between 3 and 6 hpi, and NS2 and M2 mRNAs were less abundant than NS1 and M1 mRNAs, respectively. The silencing of RED, and to a lesser extent the silencing of SMU1, reduced the accumulation of viral mRNAs (Figure 6B, square and triangle symbols, respectively, compared to circle symbols). This effect was overall more pronounced for NS2 mRNAs (Figure 6B, grey box) than for NS1, M1 and M2 mRNAs (Figure 6B, open boxes), particularly so at the latest time-point (two independent experiments in triplicates with RED-siRNAs, two-way ANOVA on logarithmic-transformed data, unweighted means, $p<0.0001$ for NS2, $p>0.0001$ for NS1, M1 and M2). As a consequence, in cells depleted for RED, the ratio of NS2 over NS1 mRNAs showed a stable 4-fold decrease at 3, 6 and 9 hpi ($p<0.001$), whereas the ratio of M2 over M1 mRNAs was reduced only 2-fold at 3 hpi ($p<0.001$) and was not affected at later time points (Figure 6C, grey bars). A similar trend was observed in cells depleted for SMU1 (Figure 6C, white bars). Overall, these data indicated that the production of spliced NS2 mRNAs was specifically impaired when RED or SMU1 were depleted.

The effect of RED or SMU1 knock-down on the accumulation of viral proteins was then examined. A549 cells treated with RED, SMU1 or control siRNAs were infected with influenza virus WSN at a m.o.i. of 5 pfu/cell. The accumulation of viral proteins was monitored by western-blot analysis of cell lysates, using antibodies for the NP, M1, M2, NS1 and NS2 proteins. The signals were quantified and normalized with respect to tubulin. As shown in Figure 7, the silencing of RED, and to a lesser extent the silencing of SMU1, reduced the accumulation of viral proteins. The ratio of NS2 over NS1 proteins was reduced at 6 and 9 hpi (5- and 3-fold, respectively, in RED-silenced cells; 3- and 2-fold, respectively, in SMU1-silenced cells), whereas the ratio of M2 over M1 protein remained stable except for a 2-fold reduction in RED-silenced cells at 6 hpi. Taken together, our data indicate that RED or SMU1 depletion induce a specific and parallel decrease of NS2 to NS1 ratio at the protein and mRNA levels.

Knock-down of RED impairs vRNP export in infected cells

One essential function of the NS2 protein is to mediate the export of neosynthetized vRNPs from the nucleus to the cytoplasm, which allows them to be transported to the cellular plasmic membrane and incorporated into virions. We thus examined whether, in cells silenced for RED, the decreased accumulation of NS2 was associated with a defect of vRNP nuclear export. A549 cells treated with RED or control siRNAs were infected with influenza virus WSN at a m.o.i. of 5 pfu/cell. At 5 hpi, the intracellular localisation of the NP protein was detected by indirect immunofluorescence. In cells treated with the control siRNA (Figure 8A, left panels), NP staining was mostly cytoplasmic, which was indicative of an efficient nuclear export of neo-synthetized vRNPs. In contrast, in cells treated with the RED siRNA (Figure 8A, right panels), the NP was detected mainly in the nucleus, most cells showing an accumulation of NP in the cortical area beneath the nuclear membrane. Scoring of NP localisation on a mean of 120 cells per experimental condition indicated that RED depletion decreased the proportion of cells in which NP was exclusively cytoplasmic, and increased the proportion of cells in which NP was exclusively nuclear (Figure 8B). A delay in vRNP export was also observed when the experiment was performed with the P908-WSN virus (Supplemental Figure S5).

Discussion

In this study we report that the cellular splicing factors RED and SMU1 are essential regulators of influenza A virus infection through the control of viral mRNA splicing. Little is known about

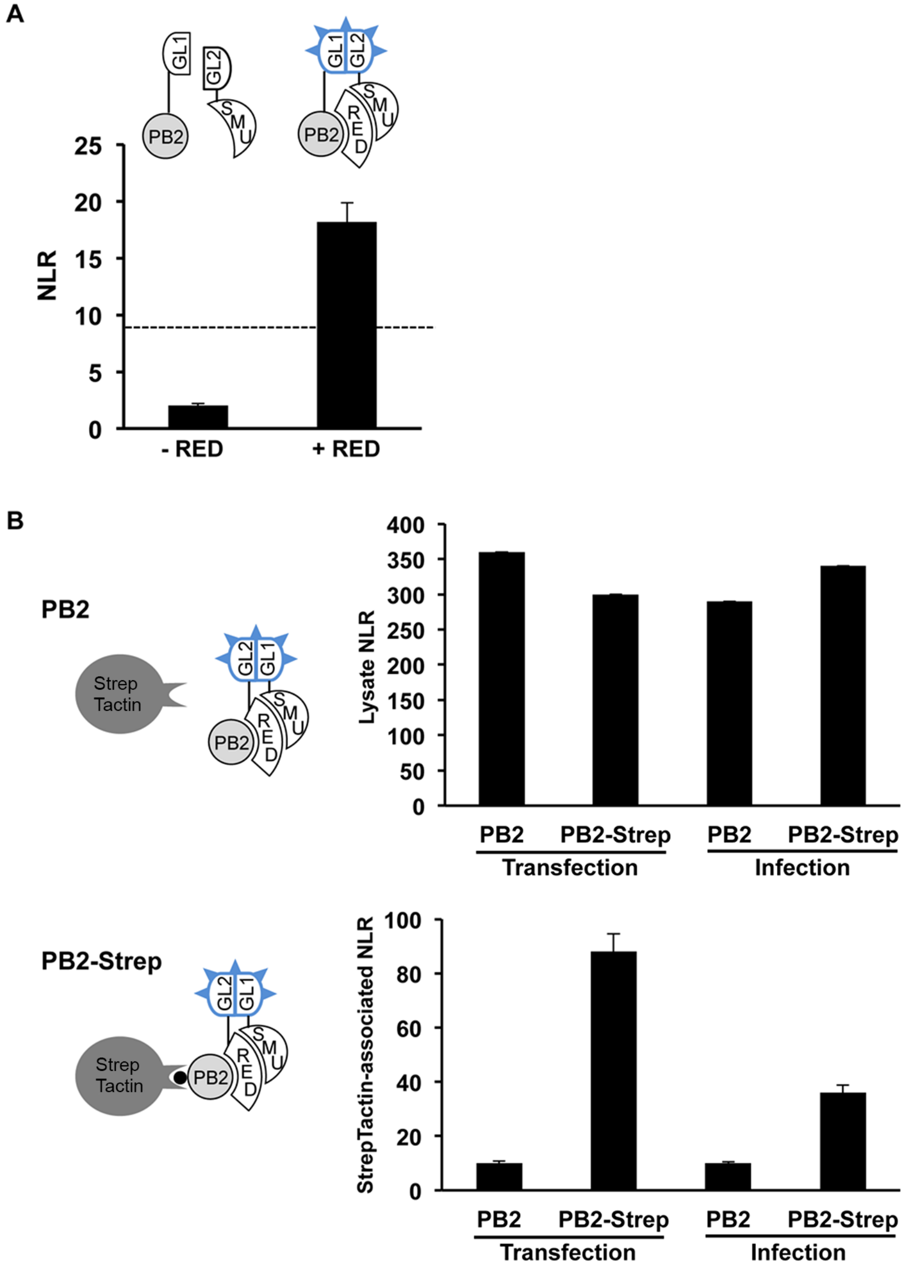

Figure 3. Detection of ternary PB2-RED-SMU1 complexes. A. The *Gaussia princeps* luciferase-based complementation assay was performed with the PB2-Gluc1 and Gluc2-SMU1 fusion proteins, in the absence or presence of over-expressed RED protein. The Normalized Luminescence Ratios (NLR) were determined as described in Figure 1A. The dashed line indicates the NLR cut-off that reduces false positive background below 2.5%, as determined using a random reference set of human proteins. The data are expressed as the mean +/− SD of triplicates and are representative of two independent experiments. **B**. The Gluc2-RED and Gluc1-SMU1 fusion proteins were co-expressed in HEK-293T cells, together with the wild-type PB2 protein or PB2-Strep fusion protein (transfection). Alternatively, HEK-293T cells co-expressing Gluc2-RED and Gluc1-SMU1 were superinfected with the rWSN or rWSN-PB2-Strep viruses (infection). Cell lysates were subjected to purification using StrepTactin beads. Control samples (cells co-expressing Gluc2-RED+Gluc1 or Gluc2+Gluc1-SMU1) were processed in parallel. Luciferase activities and NLRs were determined on an aliquot of the lysates (upper graph) and on the StrepTactin eluates (lower graph) as described in Figure 1A. The data are expressed as the mean +/− SD of triplicates and are representative of two independent experiments (infection) or a single experiment (transfection).
doi:10.1371/journal.ppat.1004164.g003

the function of the human RED and SMU1 proteins within the spliceosome, apart from the fact that a temperature-sensitive mutation in SMU1 is associated with a defect in *unc52/perlecan* pre-mRNA splicing [40], and that RED interacts with SMU1 and other spliceosomal factors in yeast two-hybrid and co-immuno-precipitation assays [31]. Highly conserved homologues of RED and SMU1 were identified in *Caenorhabditis elegans* and *Arabidopsis thaliana*, and were also found to interact with each other *in vitro* and *in vivo* [19,21]. The homologues in *Caenorhabditis elegans*, SMU2 and SMU1 (35% and 62% identity with their human RED and SMU1 counterparts, respectively) were shown to work together to regulate the splicing of specific pre-mRNAs. Indeed, mutations in SMU2 and in SMU1 resulted in the very same alterations of the splicing of *unc52* [19] and *unc73* pre-mRNA [20], which pointed to

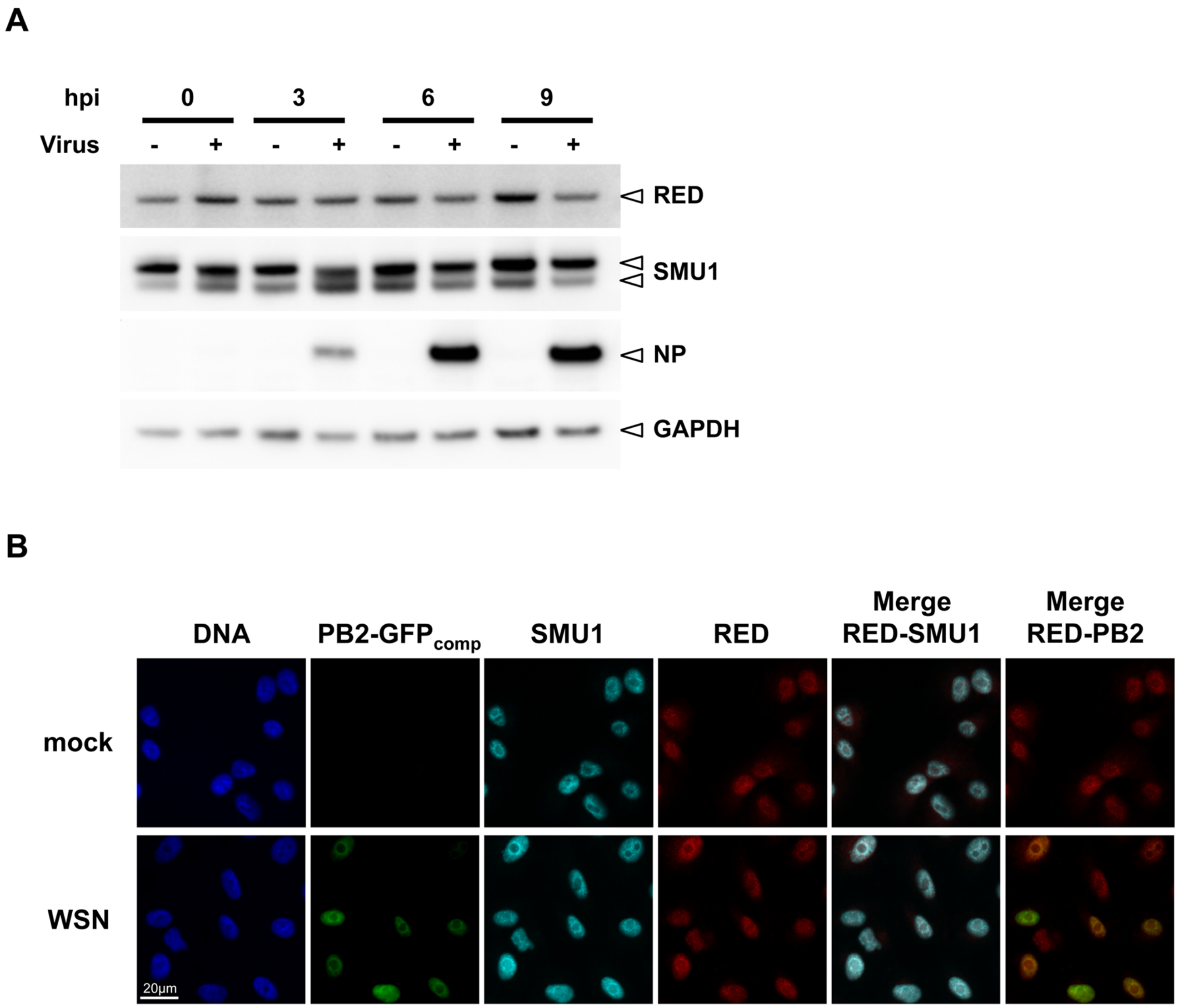

Figure 4. Steady-state levels and subcellular localisation of endogenous RED and SMU1 in infected cells. A. A549 cells were infected at a m.o.i. of 5 pfu/cell with the rWSN virus (+) or mock-infected (−). Total cell extracts were prepared at 0, 3, 6 and 9 hours post-infection (hpi), loaded on a 4–12% SDS-polyacrylamide gel and analyzed by western blotting using antibodies specific for RED, SMU1, NP and GAPDH. **B**. A549-GFP1-10 cells on coverslips were infected with the rWSN-PB2-GFP11 recombinant virus at a m.o.i. of 5 pfu/cell (WSN), or mock-infected. These conditions allow direct visualization of PB2 in infected cells by trans-complementation of GFP fragments GFP1-10 and GFP11 [38]. At 6 hpi, cells were fixed, permeabilized, and stained with an antibody specific for the RED or SMU1 protein and with Hoechst 33342. Samples were analyzed under a fluorescence microscope (Inverted Zeiss Observer Z1). Merge views corresponding to RED (red) and SMU1 (cyan), or RED and PB2-GFPcomp (green), are shown. A scale bar is shown.
doi:10.1371/journal.ppat.1004164.g004

a possible role of these two factors in selection of cryptic 5′ splice sites. Consistently, mutants of *Arabidopsis thaliana* AtSMU2 and AtSMU1 showed altered splicing of a common set of pre-mRNAs, and similar developmental abnormalities [21]. No such functional association between the human RED and SMU1 proteins had been reported so far. Here we show that indeed, along with their worm and plant homologues, the human RED and SMU1 proteins cooperate as they jointly control the splicing of NS1 pre-mRNAs during influenza virus infection. Further studies would be needed to evaluate how important is the role played by RED/SMU1 in the regulation of human pre-mRNA splicing, in particular with regard to the development of cancer and other pathologies that can result from splicing alterations [41]. Indeed, somatic mutations on RED and SMU1 are reported in the Cancer Gene Census database (COSMIC).

We show that RED and SMU1 co-purify with influenza virus polymerase in infected cells. The N-terminal domain of RED is responsible for binding to SMU1, and also to the viral polymerase subunits PB1 and PB2. No interaction between SMU1 and any of the polymerase subunit was detected, but we present evidence for the formation of PB2-RED-SMU1 ternary complexes. Overall, our protein-protein interaction mapping data indicate that RED is mediating the association of RED-SMU1 complexes with the PB2 protein. They are consistent with cross-effects of RED and SMU1 silencing observed by us and by others [19,21], which suggest that the two proteins are stabilizing each other and that a significant

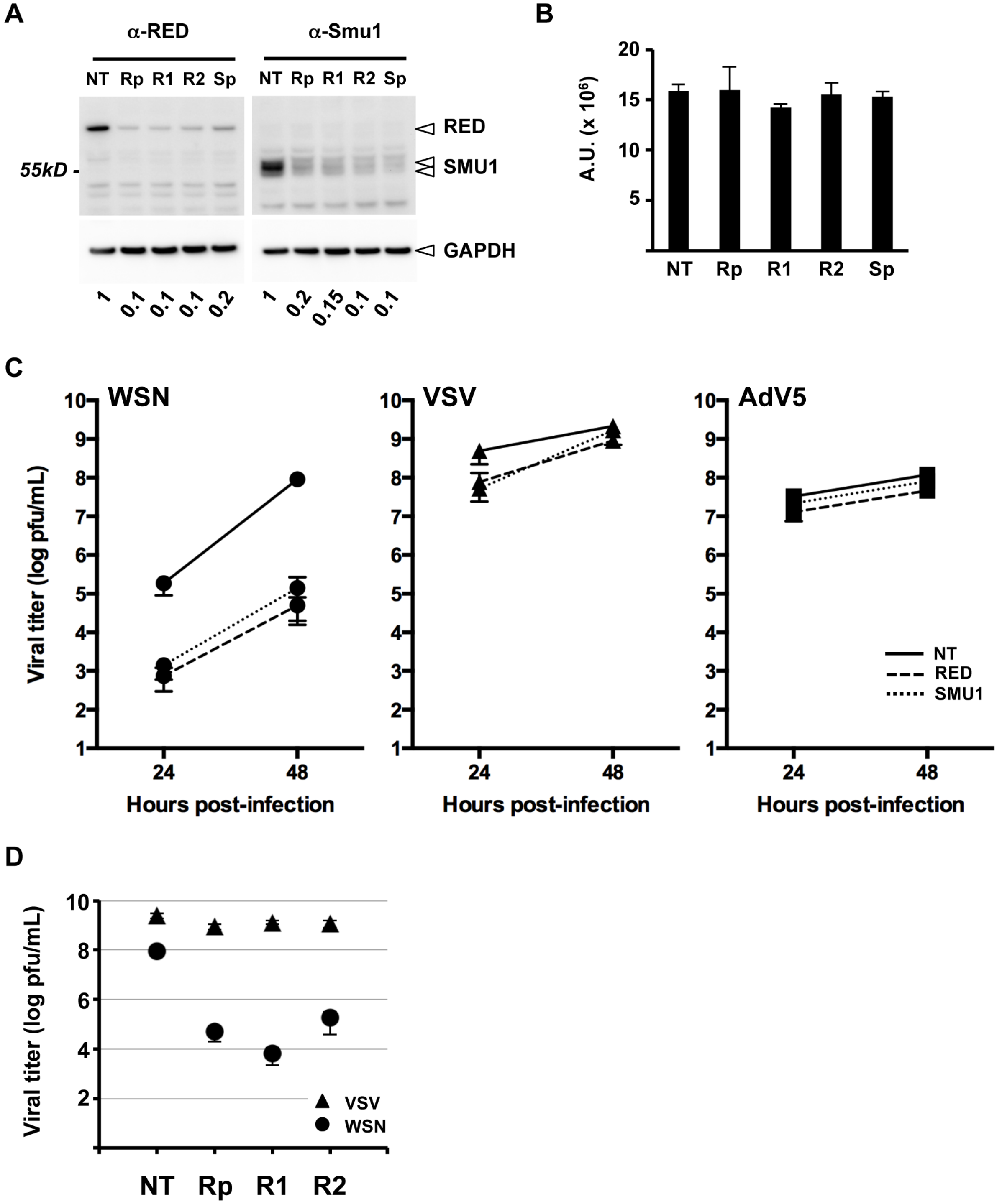

Figure 5. Effect of RED or SMU1 knock-down on influenza virus multicycle growth. A. A549 cells were transfected with control non-target siRNAs (NT), a pool of four RED siRNAs (Rp), individual RED siRNAs (R1, R2), or a pool of four SMU1 siRNAs (Sp). Total cell extracts were prepared at 36 hours post-transfection, and analyzed by western blotting using antibodies specific for RED (left upper panel), SMU1 (right upper panel) and GAPDH (lower panels). The residual levels of SMU1 and RED in silenced cells relative to control cells are indicated. The data are representative of two independent experiments. **B**. A luminescence-based cell viability assay was performed on RED and SMU1 siRNA-treated cells at 36 hours post-transfection. The data are expressed as the mean +/− SD of triplicates and are representative of two independent experiments. A.U.: arbitrary units. **C**. A549 cells were transfected with control (NT, solid lines), RED (dashed lines) or SMU1 (dotted lines) siRNAs. At 36 hours post-transfection, they were infected with WSN influenza virus (circles), VSV (triangles), or Adenovirus 5 (squares) at a m.o.i. of 10^{-3}, 10^{-4} and 6 pfu/cell, respectively. Supernatants of WSN- and VSV-infected cells were collected at 24 and 48 hours post-infection and were titrated by plaque assay. Lysates of AdV5 infected cell were prepared at 24 and 48 hpi and were titrated by immunostaining. The data are expressed as the mean +/− SD of triplicates (WSN, VSV) or duplicates (AdV5), and are representative of two (WSN, VSV) or one (AdV5) independent experiments. **D**. A549 cells were transfected with

control (NT) or with the indicated RED siRNAs. At 36 hours post-transfection, they were infected with the WSN virus (circles) or VSV (triangles) at a m.o.i. of 10^{-3} or 10^{-4} pfu/cell, respectively. Supernatants were collected at 48 hours post-infection and were titrated by plaque assay. The data are expressed as the mean +/− SD of triplicates.
doi:10.1371/journal.ppat.1004164.g005

proportion is present as functional RED-SMU1 complexes. Interestingly, in a systematic affinity tagging/purification mass spectrometry study, the RED and SMU1 proteins were found to be associated with the HIV-1 Rev protein [42], suggesting that the RED-SMU1 complex might be targeted by a larger range of viruses.

The RED protein that co-purifies with PB2-Strep in infected cell lysates is most likely associated to the viral trimeric polymerase. RED could bind to polymerases associated with RNPs, possibly a low proportion of these if the RED-polymerase interaction is stabilized by cis-acting RNA signals present in some but not all RNPs depending on their RNA component (e.g. NS *versus* other viral RNAs) and/or on their status (*e.g.* actively transcribing vRNPs *versus* vRNPs ready for export). RED might also bind to polymerases that are not incorporated into an RNP, as RED-PB2 and RED-PB1 interactions can be detected in the absence of other viral proteins or viral RNA. These free polymerases bound to RED-SMU1 could in turn modulate the splicing of viral RNAs though cooperation and oligomerization with polymerases that reside on actively transcribing RNPs. This hypothesis is in line with a recent publication showing that *trans*-activating polymerases are required for activation of resident polymerases which carry out vRNA synthesis [43].

The 5′ splice site in influenza virus NS1 pre-mRNA is around 70 nt downstream the cap structure and corresponds to the suboptimal *5′*-C A G ↓ G T A G A T-*3′* sequence, distinct from the consensus *5′*-A/C A G ↓ G T A/G A G T-*3′* sequence. It was recently demonstrated that the presence of such a weak splice site and the resulting slow accumulation of the splice product, the NS2 protein, are beneficial for the virus as they ensure a proper timing of viral replication [9]. Our observation that the NS1 pre-mRNA splicing is impaired in cells depleted for RED or SMU1, taken together with our protein-protein interaction findings, suggest a model in which the influenza virus polymerase recruits the RED-SMU1 complex to ensure recognition of the weak 5′ splice site in NS1 mRNAs, and to control this essential checkpoint of the viral life cycle. A previous study showed that the viral polymerase controls the choice of alternative 5′ splice sites in the M1 mRNA, by binding to the distal, stronger 5′ splice site and thus allowing the switch to the proximal, weaker 5′ splice site and the production of the M2 mRNA [11]. The viral polymerase thus seems to play a central role in coupling transcription and alternative splicing of the viral mRNAs. This is in line with the current view that cellular mRNA synthesis, processing and splicing are closely linked [44,45].

Our study is stressing the fact that different cellular factors and mechanisms are involved in the splicing regulation of influenza virus NS1 and M1 mRNAs. Indeed, the effect of RED-SMU1 depletion is more pronounced for NS1 mRNA splicing than for M1 mRNA splicing in WSN-infected cells. Conversely, the cellular proteins hnRNP K and NS1-BP were recently shown to regulate the splicing of M1, but not NS1 mRNAs in WSN-infected cells [18]. Whether RED and/or SMU1 bind directly and specifically to NS1 pre-mRNAs remains to be explored. An alternative hypothesis is that the recognition of NS1 mRNA is mediated by one or several cellular factors associated to the RED-SMU1 complex. Indeed, none of the RED and SMU1 proteins contains typical RNA-binding domain. In contrast, both proteins show motifs that promote the assembly of multi-protein complexes, *i.e.* coiled-coil motifs in the N-terminal domain of RED [24], two nuclear receptor binding motifs (LXXLL) and WD40 repeats in the N-terminal and C-terminal domains of SMU1, respectively (the Eukaryotic Linear Motif resource [46]). A comprehensive mapping of protein-protein interactions among human spliceosomal proteins suggested that RED and SMU1, together with MFAP1, may contribute to protein recruitment during B complex formation at an early stage of splicing [31].

Upon influenza virus infection of cells silenced for RED or SMU1, the NS2/NS1 protein ratio was decreased and the production of NS2 was strongly reduced. In agreement with the well-documented function of NS2 as an exportin, the nuclear export of neo-synthetized vRNPs was strongly impaired, which very likely contributed to the reduced production of infectious progeny virions. The NS2 protein was recently found to be involved in other aspects of the viral cycle (for a review, see [6]), including the regulation of viral RNA transcription and replication [47]. This is in agreement with our observation that the accumulation of other viral mRNAs and proteins (NS1, M1, M2, NP) was reduced at early stages of infection in cells silenced for RED or SMU1, which could be a consequence of the low levels of NS2 synthesis. In addition, distinct, non-NS2-mediated effects of RED depletion on the viral cycle can be envisioned. Indeed, beyond its function as a splicing factor, the RED protein was reported to associate to the spindle poles and to be required for mitotic progression [37]. It would thus be interesting to explore whether the viral polymerase-RED interaction is linked to the prevention of cell cycle entry into S phase [48].

The investigation of cellular pathways that are essential for influenza A virus replication, and the identification of the underlying interactions between viral and cellular components, might open the way to new antiviral strategies. Based on fundamental research on HIV-1 alternative splicing mechanisms, a small molecule was identified that interferes with the activity of the splicing factor SF2/ASF, inhibits the production of key viral proteins, and as a consequence inhibits the production of infectious particles [49]. Similarly, our findings could open the way to new antiviral strategies aimed at disrupting the interactions that exist between the viral machinery of transcription and the cellular splicing machinery to block the expression of the key NS2/NEP protein and to inhibit influenza virus multiplication.

Materials and Methods

Plasmids

The Gateway-compatible donor plasmids containing cellular ORFs were obtained from the Human ORFeome resource (hORFeome v3.1 and v5.1), except from the SMU1 plasmid which was kindly provided by Ulrich Stelzl (Max Planck Institue for Molecular Genetics, Berlin, Germany). They were transferred into the Gateway-compatible pSPICA-N2 or pSPICA-N1 destination vector [27], or into a Gateway-compatible pmCherry destination vector using the LR clonase (Invitrogen). The resulting plasmids allowed the expression of Gluc2, Gluc1 or mCherry fusion proteins. The Gateway-compatible donor plasmid containing the RED ORF was used as a template to amplify the RED, Nt-RED or Ct-RED coding sequence. The resulting amplicons, which encoded either untagged or HA-tagged RED, Nt-RED and Ct-RED depending on the oligonucleotides that were used, were

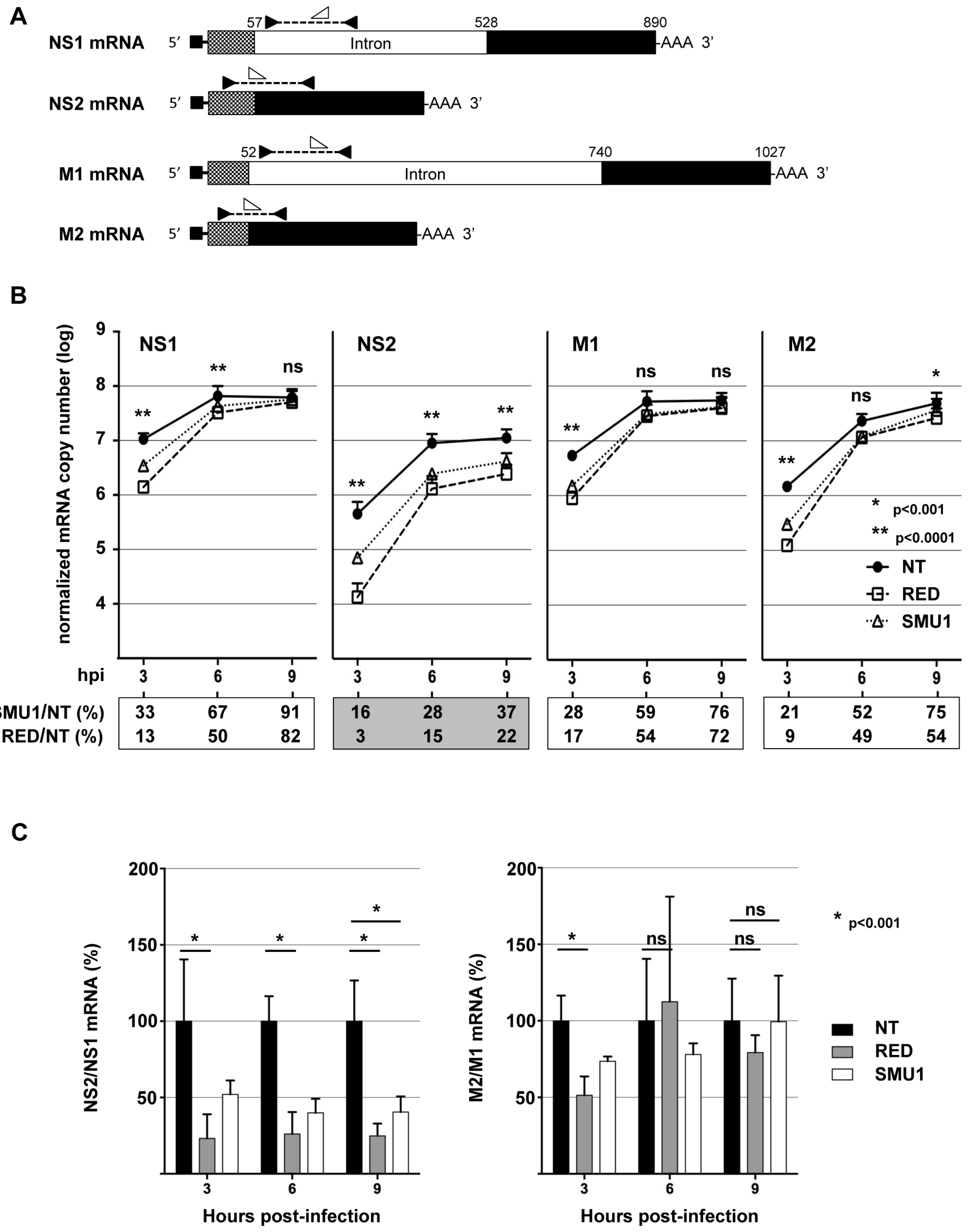

Figure 6. Effect of RED or SMU1 knock-down on the accumulation of influenza virus mRNAs. A549 cells were transfected with RED (R), SMU1 (S) or control non-target (NT) siRNAs, and were subsequently infected with the WSN virus at a m.o.i. of 5 pfu/cell. Polyadenylated RNAs were purified at 3, 6 and 9 hpi, and RT-qPCR was performed to detect specifically the viral NS1, NS2, M1 and M2 mRNAs, as well as the cellular GAPDH mRNAs. **A**. Schematic representation of the primers and probes used for RT-qPCR. The NS1, NS2, M1 and M2 mRNAs are depicted. The positions of primers and probes are indicated by black and white arrowheads, respectively, that are oriented according to the sense or antisense orientation of the oligonucleotides. **B**. The NS1, NS2, M1 and M2 mRNA copy numbers, as determined using the standard curve method and normalized with

respect to GAPDH mRNA copy numbers, are shown. The data are expressed as the mean +/− SD of two independent experiments in triplicates, except for the SMU1-siRNA-3 hpi and SMU1-siRNA-6 hpi conditions (one experiment in triplicates). For each mRNA at each time-point, the ratio (in percent) of mRNA copy number in RED- and SMU1-silenced cells to mRNA copy number in NT siRNA-treated cells are indicated below the graphs. Two-way ANOVA was performed to evaluate the effects of RED siRNA treatment. **: $p<0.0001$; *: $p<0.001$; ns: non significant ($p>0.05$). **C.** The ratios of NS2/NS1 and M2/M1 mRNA copy numbers in RED- and SMU1-silenced cells are expressed as percentages of the ratios measured in control cells (black bars: 100%). When indicated, two-way ANOVA was performed to evaluate the effect of siRNA treatment. *: $p<0.001$; ns: non significant ($p>0.05$).
doi:10.1371/journal.ppat.1004164.g006

cloned into the pCI vector (Promega) using *Xho*I and *Mlu*I restriction sites. Plasmids encoding the Gluc2-tagged RED, Nt-RED and Ct-RED were obtained by replacing the HA tag with the Gluc2 sequence. All constructs were verified by Sanger sequencing. The sequences of the oligonucleotides used for amplification and sequencing can be provided upon request. The pCMV-GFP1-10 plasmid was purchased from Sandia Biotech.

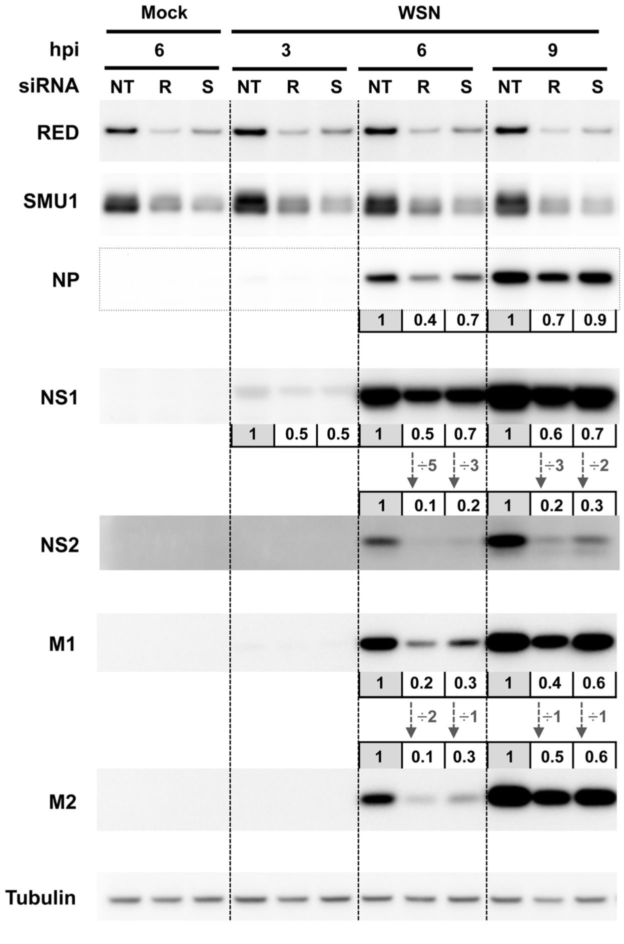

Figure 7. Effect of RED or SMU1 knock-down on the accumulation of influenza virus proteins. A549 cells were transfected with control non-target (NT), RED (R), or SMU1 (S) siRNAs, and were subsequently infected with the WSN virus at a m.o.i. of 5 pfu/cell. Total cell extracts were prepared at 0, 3, 6 and 9 hours post-infection (hpi), loaded on a 4–12% SDS-polyacrylamide gel and analyzed by western blotting using antibodies for RED, SMU1, NP, NS1, NS2, M1, M2 and tubulin. The membranes were scanned with a G-box (Syngene) and the signals were quantified using GeneTools software (Syngene) and normalized with respect to tubulin. For each viral protein at each time point, the levels in RED- and SMU1-silenced cells relative to control cells (set at an arbitrary value of 1) are indicated. The NS2/NS1 and M2/M1 ratios are indicated by vertical arrows.
doi:10.1371/journal.ppat.1004164.g007

Cells and viruses

HEK-293T, HEK-293, A549 and BSR cells were grown in complete Dulbecco's modified Eagle's medium (DMEM) supplemented with 10% fetal calf serum (FCS). MDCK cells were grown in modified Eagle's medium (MEM) supplemented with 5% FCS.

Influenza virus A/Paris/908/97 ([P908], H3N2) was isolated at the National Influenza Center at the Institut Pasteur (Paris, France). The recombinant A/WSN/33 viruses encoding a PB2-Strep [33] or a PB2-GFP11 protein [38], have been described previously. The recombinant P908-WSN and P908-Cstrep-WSN viruses have been described in [34]. VSV (Indiana strain) was kindly provided by Olivier Delmas (Institut Pasteur, Paris, France). The Adenovirus 5 was kindly provided by Puri Fortes (Universidad de Navarra, Pamplona, Spain).

Protein complementation assays

The protein complementation assay was performed as described in [27]. Briefly, HEK-293T cells seeded in 96-well white plates were co-transfected in triplicates with 100 ng of the recombinant p-Gluc2-cellular-ORF plasmids and 25 ng of the expression plasmid encoding a polymerase subunit fused to Gluc1 (pCI-PB2-Gluc1, pCI-PB1-Gluc1 or pCI-PA-Gluc1). Control cells were co-transfected either with 100 ng of empty p-Gluc2 and 25 ng of pCI-Pol-Gluc1 plasmids or with 100 ng of p-Gluc2-cellular-ORF and 50 ng of empty p-Gluc1 plasmids. In some experiments pCI-RED expression plasmid was added. The total DNA amount was adjusted at 225 ng with pCI plasmid, and transfections were performed using polyethylenimine PEI (Polysciences Inc). After 6 h of incubation, cells were lysed with 40 µL of *Renilla* lysis buffer (Promega) and the *Gaussia princeps* luciferase enzymatic activity was measured using the *Renilla* luciferase assay reagent (Promega) and a Berthold Centro XS luminometer. The Normalized Luminescence Ratios (NLRs) were calculated as follows: the luminescence activity measured in cells transfected with the p-Gluc2-cellular-ORF plasmid and with the PB2-Gluc1, PB1-Gluc1 or PA-Gluc1 plasmid (Arbitrary Units, mean of triplicates), divided by the sum of the luminescence activities measured in both control samples as described above (Arbitrary Units, mean of triplicates). The NLR cut-off value that reduces false positive background below 2.5% (NLR = 8) was determined using a random reference set of 13 randomly picked human proteins (APOOL, GYPA, NFE2L1, PLEKHA9, NXPH1, CNTN2, DBH, MDP1, GSTT1, DPYSL2, UGT3A1, NCRNA00246B, LRRC28), as described in [50].

vRNP purification on StrepTactin beads

HEK-293T ($1–2\times10^7$) cells were infected with WSN or P908/WSN recombinant viruses at a m.o.i. of 3. Six hours post-infection, cells were lysed in 0.5 ml of lysis buffer (20 mM MOPS-KOH pH 7.4, 120 mM of KCl, 0.5% Igepal), supplemented with Complete Protease Inhibitor Mixture (Roche). Cell lysates were processed and incubated wih StrepTactin beads (StrepTactin Sepharose High Performance, GE Healthcare) as described in [51]. After three washes with 1 ml of lysis buffer, protein complexes were eluted from StrepTactin beads with desthiobiotin (IBA). Purification samples were either diluted in Laemmli sample

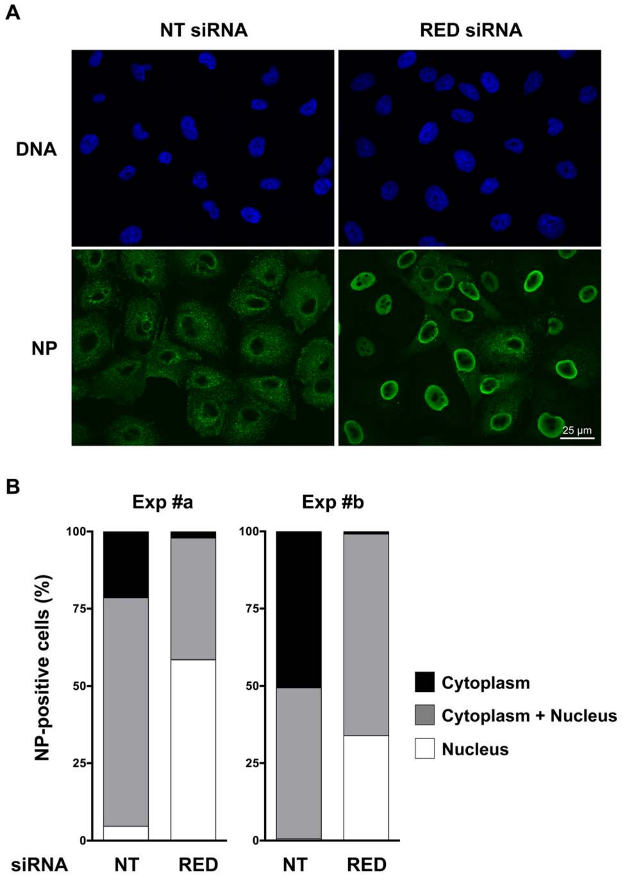

Figure 8. Effect of RED knock-down on the subcellular localization of NP in influenza virus infected cells. A549 cells were transfected with control non-target (NT) or RED siRNAs, and were subsequently infected with WSN influenza virus at a m.o.i. of 5 pfu/cell. At 5 hpi, cells were fixed, permeabilized, and stained with an antibody specific for the NP protein and with Hoechst 33342. Samples were analyzed under a fluorescence microscope (Inverted Zeiss Observer Z1). **A**. Representative images of NP localization. **B**. Percentage of cells with different NP localization. The results of two independent experiments is shown, in which 107 and 94 cells (exp #a), and 164 and 115 cells (exp #b), were scored for the NT and RED siRNA experimental condition, respectively.
doi:10.1371/journal.ppat.1004164.g008

buffer and analyzed by western-blot, or diluted in *Renilla* lysis buffer (Promega) and submitted to *Gaussia princeps* luciferase enzymatic activity measurement, using the *Renilla* luciferase assay reagent (Promega) and a Berthold Centro XS luminometer.

siRNA transfection and infection assays

Small interfering RNAs (siRNAs) targeting RED were purchased from Dharmacon (ON-TARGETplus SMARTpool or individual siRNAs). Non-target siRNA (ON-TARGETplus Non-targeting Control pool, Dharmacon) was used as a negative control. A549 cells were transfected with 25 nM of siRNA using the DharmaFECT1 transfection reagent (Dharmacon) and were plated in 96-well plates (2×10^4 cells per well) or in 12-well plates (2×10^5 cells per well). Cell viability was determined by assaying total intracellular ATP with the CellTiter-Glo Luminescent Viability Assay kit (Promega). Down-regulation of siRNA-targeted genes was evaluated by RT-qPCR using Maxima First Strand cDNA Synthesis kit and Solaris qPCR Expression Assays and Master Mix (Thermo Scientific), or by western blot. For multicycle growth assays and single cycle infection assays, cells were infected at 36 h post-transfection at a low and a high moi, respectively. After 1 h of viral adsorption, the cells were incubated at 37°C in DMEM supplemented with 2% FCS. The production of infectious virus particles in the culture supernatant (WSN, VSV) or in the infected cells (AdV5) was determined using a plaque assay [52] on MDCK (WSN) and BSR cells (VSV), or an immuno-staining assay on HEK-293 cells (AdV5) as described [53]. Total cell lysates prepared at different time points by direct lysis in Laemmli buffer were analyzed by western blotting.

Antibodies and western blot assays

Western blots were performed as described earlier [54]. The membranes were incubated with primary antibodies directed against RED or SMU1 (Santa Cruz), A/PR/8/34 virions [55], NS1 (kindly provided by Daniel Marc, INRA-Tours, France), NS2 (kindly provided by Florence Baudin, EMBL-Heidelberg, Germany), M1 (clone GA2B, Pierce), M2 (clone 14C2, Pierce), GAPDH (Pierce), tubulin (Calbiochem), the HA tag (clone 16B12, Covance), the Gaussia luciferase (New England Biolabs), with peroxidase-conjugated Streptavidin (IBA) and peroxidase-conjugated secondary antibodies (GE Healthcare), and with the ECL 2 substrate (Pierce). The membranes were scanned in a G-Box (Syngene), the chemiluminescence was acquired and quantified with the GeneSnap and GeneTools softwares (SynGene), respectively.

Reverse transcription - quantitative PCR assays

For RT-qPCR analysis of viral mRNAs, infected A549 cells were first submitted to total RNA extraction, using the RNeasy mini kit (Qiagen). Total RNA was resuspended in nuclease-free water, and isolation of polyA+ RNAs was performed using the Oligotex kit (Qiagen). Total and polyA+ RNAs were quantified by measuring the absorbance at 260 nm with the NanoDrop Lite (Thermo Scientific). The polyA+ RNA fractions represented about 10% of the amounts of total RNA. Reverse transcription was performed on 20 ng of polyA+ RNA, using the Maxima First Strand cDNA Synthesis kit which includes a mixture of oligo-dT and random hexamer primers (Thermo Scientific), in a final volume of 20 µl. Negatives controls, *i.e.* reactions set up in the absence of the reverse transcriptase enzyme, were run concurrently for all samples. Real-time PCR was performed on 2 µl of a 1:10 dilution of the reverse-transcription reaction, using the Solaris qPCR Master Mix (Thermo Scientific), sets of primers and probe as provided in the Solaris qPCR Gene Expression Assays (Thermo Scientific), and a Light Cycler 480 (Roche). For GAPDH, RED and SMU1 transcripts, primers and probes were designed and inventoried by Thermo Scientific. For M1, M2, NS1 and NS2 transcripts, specific primers and probes were designed by us using the CLC Main Workbench 6.8.3 and primer3 softwares and were made to order. The corresponding sequences are indicated in Methods S1. The qPCR protocol consisted of an initial activation step of 15 min at 95°C, 45 amplification cycles with 15 sec at 95°C/30 sec at 55°C/30 sec at 60°C and a final cooling step of 30 sec at 40°C. The cycle thresholds (Ct) were determined by second derivative quantification using the analytical LightCycler 480 Software, release 1.5 (Roche). The Ct were either analyzed using the ΔΔCt method [56] or converted into numbers of cDNA copies using the standard curve method. In the latter case, the calibration curves were based on serial dilutions of a pDON223-GAPDH plasmid (Human ORFeome resource) or of pUC57 plasmids containing synthetic sequences corresponding to the M1, M2, NS1 and NS2 cDNAs (GenScript). These calibration curves

were also used to assess the specificity of each set of primers and probe.

Indirect immunofluorescence assays

A549-GFP1-10 or A549 cells on coverslips were infected with the rWSN-PB2-GFP11 or rWSN recombinant virus, respectively, at a m.o.i. of 5 pfu/cell, incubated with DMEM supplemented with 2% FCS for 0–9 hours, fixed with PBS-4% paraformaldehyde for 20 min, and permeabilized with PBS-0.1% Triton X100 for 10 min. A549-GFP1-10 cells were incubated with a mixture of anti-RED and anti-SMU1 antibodies (Santa Cruz, diluted 1/50), and then with a mixture of DyeLight633-coupled anti-rabbit and DyeLight550 anti-mouse IgG secondary antibodies (Pierce, diluted 1/500) with Hoechst 33342 (Invitrogen, diluted to 1 µg/ml). A549 cells were incubated with a mixture of anti-RED and anti-NP (Argene, diluted 1/200) antibodies, and then with a mixture of AF555-coupled anti-rabbit and AF488 anti-mouse IgG secondary antibody (Invitrogen, diluted 1/500) with Hoechst 33342. HEK-293T cells on poly-D-lysine coated coverslips were co-transfected with 1 µg of pCMV-GFP1-10 and 1 µg of the pCI-mCherry-RED, pCI-mCherry or control pCI plasmid using the Fugene-HD transfection reagent. At 24 hours post-transfection, they were infected with the rWSN-PB2-GFP11 recombinant virus at a m.o.i. of 5 pfu/cell, incubated with DMEM supplemented with 2% FCS for 6 hours, fixed with PBS-4% paraformaldehyde for 20 min, and stained with Hoechst 33342. The samples were mounted in ProLong Gold mounting medium (Invitrogen) and analyzed under a fluorescence microscope (Inverted Zeiss Observer Z1 with HBO lamp used at 50% level) using ×40 and ×63 oil immersion objective lenses. Images were acquired with an AxioCam MRm camera and the Axiovision 4.6.3 software (Zeiss) before being exported as .tif files.

Supporting Information

Figure S1 Co-purification of RED and SMU1 with P908 virus polymerase in infected cells. A. Co-purification of the endogenous RED and SMU1 proteins with the viral polymerase in infected cells. HEK-293T cells were infected at a m.o.i. of 5 with recombinant P908-WSN (W) or P908-Cstrep-WSN (S) viruses and incubated at 37°C for 6 hours. Whole-cell lysates were prepared and a fraction was incubated with StrepTactin beads as described in Material and Methods. Protein complexes were eluted, loaded on a 4–12% SDS-polyacrylamide gel and analyzed by western blotting using either StrepTactin to detect the PB2-Strep protein (upper panel) or an antibody specific for the RED or SMU1 protein (middle and lower panels). * and ** : non-specific detection of the PB2 and NP protein, respectively, as inferred from previous experiments [33]. **B**. Co-purification of the viral polymerase and recombinant, HA-tagged polypeptides corresponding to full-length or truncated forms of the RED protein. HEK-293T cells were transfected with the HA-RED, HA-Nt-RED or HA-Ct-RED expression plasmids, or mock-transfected with the pCI plasmid (−). 24 hours post-transfection, they were infected at a m.o.i. of 5 with recombinant P908-WSN (W) or P908-Cstrep-WSN (S) viruses and incubated at 37°C for 6 hours. Whole-cell lysates were prepared and a fraction was incubated with StrepTactin beads as described in Material and Methods. Protein complexes were eluted, loaded on a 4–12% SDS-polyacrylamide gel and analyzed by western blotting using either StrepTactin to detect the PB2-Strep protein (upper panel) or a monoclonal antibody specific for the HA tag (lower panel). **: non-specific detection of the NP protein, as inferred from western blot analysis using an anti-NP antibody. The bands detected in the PB2-Strep panel at 70 kD and the faster migrating band (present only in lysates) were also detected in mock-infected cells (data not shown).
(TIF)

Figure S2 Immunofluorescence detection of RED and SMU1 in A549 cells treated with RED or SMU1 siRNAs. A549 cells were transfected with control non-target (NT), RED, or SMU1 siRNAs. At 41 hours post-transfection, cells were fixed, permeabilized, and stained with Hoechst 33342 and with an antibody specific for the RED (upper panels) or SMU1 protein (lower panels). Samples were analyzed under a fluorescence microscope (Inverted Zeiss Observer Z1).
(TIF)

Figure S3 Relocalisation of PB2 with over-expressed mCherry-RED in infected cells. HEK-293T cells on coverslips were transfected with the pCMV-GFP1-10 plasmid together with the pCI-mCherry-RED, pCI-mCherry or control pCI plasmid, as indicated. At 24 hours post-transfection, they were infected with the rWSN (PB2-wt) or rWSN-PB2-GFP11 (PB2-GFP11) recombinant virus at a m.o.i. of 5 pfu/cell, or mock-infected. Cells were fixed at 6 hpi and they were stained with Hoechst 33342. Samples were analyzed under a fluorescence microscope (Inverted Zeiss Observer Z1). A merge of the signals corresponding to Hoechst-stained DNA (blue), PB2-GFPcomp (green) and mCherry-RED (red) is shown. A scale bar is shown.
(TIF)

Figure S4 Quantification of RED and SMU1 mRNAs upon treatment of A549 cells with RED or SMU1 siRNAs. A549 cells were transfected with control siRNAs (NT), or with pools of four RED or SMU1 siRNAs. Total RNA was prepared at 36 hours post-transfection, polyA+ RNAs were isolated, and RT-qPCR was performed using primers and probes specific for RED or SMU1. The levels of RED and SMU1 mRNAs in RED/SMU1 siRNA-treated cells compared to control cells were determined using the $\Delta\Delta$Ct method. The data are expressed as the mean +/− SD of quadruplicates.
(TIF)

Figure S5 Effect of RED knock-down on the subcellular localisation of NP in P908-WSN influenza virus infected cells. A549 cells were transfected with control non-target (NT) or RED siRNAs, and were subsequently infected with the P908-WSN influenza virus at a m.o.i. of 5 pfu/cell. At 5 hpi, cells were fixed, permeabilized, and stained with an antibody specific for the NP protein and with Hoechst 33324. Samples were analyzed under a fluorescence microscope (Inverted Zeiss Observer Z1). **A**. Representative images of NP localization. **B**. Percentage of cells with different NP localization, based on the scoring of 145 and 132 cells for the NT and RED siRNA experimental condition, respectively.
(TIF)

Methods S1 Supporting information is provided on the yeast two-hybrid-plus-one assay, indirect immunofluorescence assays and reverse transcription-quantitative PCR assays.
(DOCX)

Table S1 Viral-human protein-protein interactions detected in the yeast two-hybrid-plus-one and the yeast two-hybrid assays.
(XLSX)

Acknowledgments

We are very grateful to J. Ortin and S. Landera-Buenos for helpful discussions, and for providing advice and materials for the AdV5 experiment. We thank J.F. Bureau for assistance with statistical analysis, A. Komarova for advice on purification experiments, U. Steltz, P. Fortes, G. Brownlee, F. Baudin, D. Marc, and O. Delmas for providing materials, P. Cassonnet and C. Diot for assistance with some experiments, C. Diot and C. Barbezange for critical reading of the manuscript, and S. van der Werf for her support. We thank Emmanuelle Perret and colleagues at the Plateforme d'Imagerie Dynamique, Institut Pasteur, Paris, for assistance with image data acquisition.

Author Contributions

Conceived and designed the experiments: GF CC SM CD AT POV YJ NN. Performed the experiments: GF CC SM AT POV YJ NN. Analyzed the data: GF CC SM CD AT POV YJ NN. Contributed reagents/materials/analysis tools: SM POV YJ NN. Wrote the paper: GF NN.

References

1. Resa-Infante P, Jorba N, Coloma R, Ortin J (2011) The influenza virus RNA synthesis machine: advances in its structure and function. RNA Biol 8: 207–215.
2. York A, Fodor E (2013) Biogenesis, assembly and export of viral messenger ribonucleoproteins in the influenza A virus infected cell. RNA biology 10: 1274–1282.
3. Shih SR, Suen PC, Chen YS, Chang SC (1998) A novel spliced transcript of influenza A/WSN/33 virus. Virus genes 17: 179–183.
4. Wise HM, Hutchinson EC, Jagger BW, Stuart AD, Kang ZH, et al. (2012) Identification of a novel splice variant form of the influenza A virus M2 ion channel with an antigenically distinct ectodomain. PLoS pathogens 8: e1002998.
5. Hale BG, Randall RE, Ortin J, Jackson D (2008) The multifunctional NS1 protein of influenza A viruses. The Journal of general virology 89: 2359–2376.
6. Paterson D, Fodor E (2012) Emerging roles for the influenza A virus nuclear export protein (NEP). PLoS pathogens 8: e1003019.
7. Selman M, Dankar SM, Forbes NE, Jia JJ, Brown EG (2012) Adaptive mutation in influenza A virus non-structural gene is linked to host switching and induces a novel protein by alternative splicing. Emerging Microbes & Infections 1: e42.
8. Chiang C, Chen GW, Shih SR (2008) Mutations at alternative 5′ splice sites of M1 mRNA negatively affect influenza A virus viability and growth rate. Journal of virology 82: 10873–10886.
9. Chua MA, Schmid S, Perez JT, Langlois RA, Tenoever BR (2013) Influenza A virus utilizes suboptimal splicing to coordinate the timing of infection. Cell reports 3: 23–29.
10. Priore SF, Kierzek E, Kierzek R, Baman JR, Moss WN, et al. (2013) Secondary structure of a conserved domain in the intron of influenza A NS1 mRNA. PLoS One 8: e70615.
11. Shih SR, Nemeroff ME, Krug RM (1995) The choice of alternative 5′ splice sites in influenza virus M1 mRNA is regulated by the viral polymerase complex. Proceedings of the National Academy of Sciences of the United States of America 92: 6324–6328.
12. Robb NC, Fodor E (2012) The accumulation of influenza A virus segment 7 spliced mRNAs is regulated by the NS1 protein. The Journal of general virology 93: 113–118.
13. Robb NC, Jackson D, Vreede FT, Fodor E (2010) Splicing of influenza A virus NS1 mRNA is independent of the viral NS1 protein. The Journal of general virology 91: 2331–2340.
14. Garaigorta U, Ortin J (2007) Mutation analysis of a recombinant NS replicon shows that influenza virus NS1 protein blocks the splicing and nucleo-cytoplasmic transport of its own viral mRNA. Nucleic acids research 35: 4573–4582.
15. Stertz S, Shaw ML (2011) Uncovering the global host cell requirements for influenza virus replication via RNAi screening. Microbes Infect 13: 516–525.
16. Watanabe T, Watanabe S, Kawaoka Y (2010) Cellular networks involved in the influenza virus life cycle. Cell Host Microbe 7: 427–439.
17. Shih SR, Krug RM (1996) Novel exploitation of a nuclear function by influenza virus: the cellular SF2/ASF splicing factor controls the amount of the essential viral M2 ion channel protein in infected cells. The EMBO journal 15: 5415–5427.
18. Tsai PL, Chiou NT, Kuss S, Garcia-Sastre A, Lynch KW, et al. (2013) Cellular RNA binding proteins NS1-BP and hnRNP K regulate influenza A virus RNA splicing. PLoS pathogens 9: e1003460.
19. Spartz AK, Herman RK, Shaw JE (2004) SMU-2 and SMU-1, Caenorhabditis elegans homologs of mammalian spliceosome-associated proteins RED and fSAP57, work together to affect splice site choice. Molecular and cellular biology 24: 6811–6823.
20. Dassah M, Patzek S, Hunt VM, Medina PE, Zahler AM (2009) A genetic screen for suppressors of a mutated 5′ splice site identifies factors associated with later steps of spliceosome assembly. Genetics 182: 725–734.
21. Chung T, Wang D, Kim CS, Yadegari R, Larkins BA (2009) Plant SMU-1 and SMU-2 homologues regulate pre-mRNA splicing and multiple aspects of development. Plant physiology 151: 1498–1512.
22. Arranz R, Coloma R, Chichon FJ, Conesa JJ, Carrascosa JL, et al. (2012) The structure of native influenza virion ribonucleoproteins. Science 338: 1634–1637.
23. Coloma R, Valpuesta JM, Arranz R, Carrascosa JL, Ortín J, et al. (2009) The structure of a biologically active influenza virus ribonucleoprotein complex. PLoS Pathog 5: e1000491.
24. Assier E, Bouzinba-Segard H, Stolzenberg MC, Stephens R, Bardos J, et al. (1999) Isolation, sequencing and expression of RED, a novel human gene encoding an acidic-basic dipeptide repeat. Gene 230: 145–154.
25. Neubauer G, King A, Rappsilber J, Calvio C, Watson M, et al. (1998) Mass spectrometry and EST-database searching allows characterization of the multi-protein spliceosome complex. Nature genetics 20: 46–50.
26. Zhou Z, Licklider LJ, Gygi SP, Reed R (2002) Comprehensive proteomic analysis of the human spliceosome. Nature 419: 182–185.
27. Cassonnet P, Rolloy C, Neveu G, Vidalain PO, Chantier T, et al. (2011) Benchmarking a luciferase complementation assay for detecting protein complexes. Nat Methods 8: 990–992.
28. Deng T, Engelhardt OG, Thomas B, Akoulitchev AV, Brownlee GG, et al. (2006) Role of ran binding protein 5 in nuclear import and assembly of the influenza virus RNA polymerase complex. J Virol 80: 11911–11919.
29. Gabriel G, Herwig A, Klenk HD (2008) Interaction of polymerase subunit PB2 and NP with importin alpha1 is a determinant of host range of influenza A virus. PLoS Pathog 4: e11.
30. Huarte M, Sanz-Ezquerro JJ, Roncal F, Ortín J, Nieto A (2001) PA subunit from influenza virus polymerase complex interacts with a cellular protein with homology to a family of transcriptional activators. J Virol 75: 8597–8604.
31. Hegele A, Kamburov A, Grossmann A, Sourlis C, Wowro S, et al. (2012) Dynamic protein-protein interaction wiring of the human spliceosome. Molecular cell 45: 567–580.
32. Chase GP, Rameix-Welti MA, Zvirbliene A, Zvirblis G, Gotz V, et al. (2011) Influenza virus ribonucleoprotein complexes gain preferential access to cellular export machinery through chromatin targeting. PLoS Pathog 7: e1002187.
33. Ge X, Rameix-Welti MA, Gault E, Chase G, dos Santos Afonso E, et al. (2011) Influenza virus infection induces the nuclear relocalization of the Hsp90 co-chaperone p23 and inhibits the glucocorticoid receptor response. PLoS One 6: e23368.
34. Rameix-Welti MA, Tomoiu A, Dos Santos Afonso E, van der Werf S, Naffakh N (2009) Avian Influenza A virus polymerase association with nucleoprotein, but not polymerase assembly, is impaired in human cells during the course of infection. Journal of virology 83: 1320–1331.
35. Krief P, Augery-Bourget Y, Plaisance S, Merck MF, Assier E, et al. (1994) A new cytokine (IK) down-regulating HLA class II: monoclonal antibodies, cloning and chromosome localization. Oncogene 9: 3449–3456.
36. Cao LX, Le Bousse-Kerdiles MC, Clay D, Oshevski S, Jasmin C, et al. (1997) Implication of a new molecule IK in CD34+ hematopoietic progenitor cell proliferation and differentiation. Blood 89: 3615–3623.
37. Yeh PC, Yeh CC, Chen YC, Juang YL (2012) RED, a spindle pole-associated protein, is required for kinetochore localization of MAD1, mitotic progression, and activation of the spindle assembly checkpoint. The Journal of biological chemistry 287: 11704–11716.
38. Avilov SV, Moisy D, Munier S, Schraidt O, Naffakh N, et al. (2012) Replication-competent influenza A virus that encodes a split-green fluorescent protein-tagged PB2 polymerase subunit allows live-cell imaging of the virus life cycle. J Virol 86: 1433–1448.
39. Valcarcel J, Portela A, Ortin J (1991) Regulated M1 mRNA splicing in influenza virus-infected cells. The Journal of general virology 72 (Pt 6): 1301–1308.
40. Sugaya K, Hongo E, Ishihara Y, Tsuji H (2006) The conserved role of Smu1 in splicing is characterized in its mammalian temperature-sensitive mutant. Journal of cell science 119: 4944–4951.
41. Cooper TA, Wan L, Dreyfuss G (2009) RNA and disease. Cell 136: 777–793.
42. Jager S, Cimermancic P, Gulbahce N, Johnson JR, McGovern KE, et al. (2012) Global landscape of HIV-human protein complexes. Nature 481: 365–370.
43. York A, Hengrung N, Vreede FT, Huiskonen JT, Fodor E (2013) Isolation and characterization of the positive-sense replicative intermediate of a negative-strand RNA virus. Proceedings of the National Academy of Sciences of the United States of America 110: E4238–4245.
44. Close P, East P, Dirac-Svejstrup AB, Hartmann H, Heron M, et al. (2012) DBIRD complex integrates alternative mRNA splicing with RNA polymerase II transcript elongation. Nature 484: 386–389.
45. Montes M, Becerra S, Sanchez-Alvarez M, Sune C (2012) Functional coupling of transcription and splicing. Gene 501: 104–117.
46. Dinkel H, Van Roey K, Michael S, Davey NE, Weatheritt RJ, et al. (2013) The eukaryotic linear motif resource ELM: 10 years and counting. Nucleic acids research 42: D259–D266.
47. Robb NC, Smith M, Vreede FT, Fodor E (2009) NS2/NEP protein regulates transcription and replication of the influenza virus RNA genome. The Journal of general virology 90: 1398–1407.

48. He Y, Xu K, Keiner B, Zhou J, Czudai V, et al. (2010) Influenza A virus replication induces cell cycle arrest in G0/G1 phase. Journal of virology 84: 12832–12840.
49. Bakkour N, Lin YL, Maire S, Ayadi L, Mahuteau-Betzer F, et al. (2007) Small-molecule inhibition of HIV pre-mRNA splicing as a novel antiretroviral therapy to overcome drug resistance. PLoS pathogens 3: 1530–1539.
50. Munier S, Rolland T, Diot C, Jacob Y, Naffakh N (2013) Exploration of binary virus-host interactions using an infectious protein complementation assay. Molecular and Cellular Proteomics 12: 2845–2855.
51. Komarova AV, Combredet C, Meyniel-Schicklin L, Chapelle M, Caignard G, et al. (2011) Proteomic analysis of virus-host interactions in an infectious context using recombinant viruses. Molecular and Cellular Proteomics 10: M110.007443.
52. Matrosovich M, Matrosovich T, Garten W, Klenk HD (2006) New low-viscosity overlay medium for viral plaque assays. Virol J 3: 63.
53. Aparicio O, Razquin N, Zaratiegui M, Narvaiza I, Fortes P (2006) Adenovirus virus-associated RNA is processed to functional interfering RNAs involved in virus production. Journal of virology 80: 1376–1384.
54. Moisy D, Avilov SV, Jacob Y, Laoide BM, Ge X, et al. (2012) HMGB1 protein binds to influenza virus nucleoprotein and promotes viral replication. Journal of virology 86: 9122–9133.
55. Vignuzzi M, Gerbaud S, van der Werf S, Escriou N (2001) Naked RNA immunization with replicons derived from poliovirus and Semliki Forest virus genomes for the generation of a cytotoxic T cell response against the influenza A virus nucleoprotein. J Gen Virol 82: 1737–1747.
56. Livak KJ, Schmittgen TD (2001) Analysis of relative gene expression data using real-time quantitative PCR and the 2(-Delta Delta C(T)) Method. Methods 25: 402–408.
